# Analytical Characterization and Stability Assessment of RNA-Based Vaccines

**DOI:** 10.3390/pharmaceutics18070790

**Published:** 2026-06-27

**Authors:** Hadi M. Alasmari, Shouq F. Alghannam, Hassan A. Al-Moammar, Dimah K. Alrabiah, Seham S. Al-Harthy, Essam J. Alyamani, Sami A. Alyahya, Mohammad Alkhrayef, Mohannad Fallatah, Samiyah Al-Khaldi, Abdulmalek T. Algarni, Yahya F. Jamous, Ahmad M. Aldossary

**Affiliations:** 1 Wellness and Preventive Medicine Institute, Health Sector, King Abdulaziz City for Science and Technology (KACST), Riyadh 11442, Saudi Arabia; hasmari@kacst.gov.sa (H.M.A.); salghannam@kacst.gov.sa (S.F.A.); halmuammar@kacst.gov.sa (H.A.A.-M.); dalrabiah@kacst.gov.sa (D.K.A.); ssalharthy@kacst.gov.sa (S.S.A.-H.); eyamani@kacst.gov.sa (E.J.A.); atalgarni@kacst.gov.sa (A.T.A.); 2 Disability Research Institute, Health Sector, King Abdulaziz City for Science and Technology (KACST), Riyadh 11442, Saudi Arabia; mkhuryef@kacst.gov.sa; 3 Advanced Diagnostics and Therapeutics Institute, Health Sector, King Abdulaziz City for Science and Technology (KACST), Riyadh 12354, Saudi Arabia; mfallatah@kacst.gov.sa; 4 Applied Genomics Technologies Institute, Health Sector, King Abdulaziz City for Science and Technology (KACST), Riyadh 11442, Saudi Arabia; salkhaldi@kacst.gov.sa

**Keywords:** RNA vaccines, lipid nanoparticles (RNA-LNPs), analytical characterization, critical quality attributes (CQAs), mRNA stability, encapsulation efficiency, potency assays

## Abstract

Ribonucleic acid-based vaccines have emerged as one of the most significant advances in modern vaccine development, demonstrating remarkable clinical success and enabling rapid responses to emerging infectious diseases. Despite their therapeutic potential, the development of these vaccines remains challenging because of the inherent instability of ribonucleic acid molecules, their susceptibility to degradation, and the complexity of formulation design. Ensuring product quality, stability, and biological performance therefore requires comprehensive analytical characterization throughout development, manufacturing, storage, and quality control. This review provides a comprehensive overview of current analytical strategies used to evaluate ribonucleic acid-based vaccine formulations. Key analytical approaches for assessing molecular integrity, purity, encapsulation efficiency, particle morphology, size distribution, surface characteristics, structural attributes, and biological potency are discussed. The review also examines the influence of formulation composition, lipid nanoparticle design, manufacturing processes, and storage conditions on vaccine stability and performance. In addition, major degradation pathways, critical quality attributes, and analytical challenges associated with quality assessment are highlighted. Furthermore, current regulatory considerations and limitations of existing analytical methodologies are discussed, particularly the challenges associated with establishing robust relationships between physicochemical properties and biological efficacy. The review emphasizes the importance of integrated multi-method analytical approaches for comprehensive characterization and quality assurance. Continued advances in analytical technologies and standardization efforts will be essential for supporting the development of safe, effective, and stable ribonucleic acid-based vaccines and for facilitating their broader pharmaceutical applications.

## 1. Introduction

Over the past decades, the concept of RNA-based therapeutics has evolved significantly. The ability of in vitro transcribed mRNA to drive protein expression in mammalian cells was shown in early studies, paving the way for RNA-based therapeutics [1]. In the 1990s, additional studies demonstrated that injected mRNA can be translated in vivo, enabling protein expression in animal tissues. These findings suggested that RNA could serve as a platform for vaccines or gene therapy. The COVID-19 pandemic in the early 2020s was a turning point in RNA therapeutics. The rapid development and demonstrated clinical efficacy of mRNA vaccines demonstrated the global applicability of RNA-based technologies. This success led to increased research and development efforts in RNA-based therapeutic strategies in the pharmaceutical industry, marking a new era in RNA therapeutics [2,3].

### 1.1. Emergence of RNA-Based Vaccines as Advanced Pharmaceutical Platforms

Building on these advances, RNA-based vaccines are now recognized as one of the most promising next-generation pharmaceutical platforms. Unlike conventional vaccines that rely on attenuated pathogens or recombinant protein antigens, RNA vaccines deliver genetic instructions that enable host cells to produce antigenic proteins internally. Intracellular antigen production induces both humoral and cellular immune responses. Recent advances in nanotechnology, particularly lipid-based nanoparticles, have provided an effective strategy for delivering mRNA to target tissues [2,4,5].

Lipid nanoparticles (LNPs) are the most clinically advanced and widely adopted delivery platform, but other delivery technologies are also actively pursued, including polymeric nanoparticles, lipid–polymer hybrid systems, nanoemulsions, exosomes, virus-like particles, and peptide-based delivery systems. These platforms have their own advantages and may further increase the therapeutic use of RNA-based vaccines [2,4].

RNA vaccine platforms offer several advantages over traditional vaccine technologies. These include rapid antigen design, flexible manufacturing processes, and the ability to quickly adapt vaccine sequences to emerging microbial variants. Furthermore, RNA vaccine platforms eliminate the need for pathogen cultivation, which eases biosafety concerns and simplifies production. Such advantages have fostered the rapid development of RNA vaccines for a variety of infectious diseases. Beyond COVID-19, RNA vaccine platforms have been investigated for influenza, rabies, Zika virus infection, Ebola, HIV, and cytomegalovirus (CMV), as well as for cancer immunotherapy, highlighting the broad therapeutic potential of RNA-based technologies [6,7]. mRNA vaccines have demonstrated significant potential in oncology as personalized cancer vaccines based on patient-specific tumor antigens encoded by mRNA, which trigger specialized immune responses against cancer cells [4,6,7]. Recent advances in RNA engineering have expanded its therapeutic applications, particularly in vaccine development and gene regulation. Besides mRNA vaccines, RNA-based therapeutic approaches based on modalities like small interfering RNA (siRNA), antisense oligonucleotides (ASOs), or self-amplifying RNA (saRNA) platforms are also in the pipeline [4,8].

RNA-based vaccines, despite their advantages, have a few challenges: they can be degraded by ribonuclease-mediated degradation, delivery issues due to their large size and negative charge, and may be immune-activated in an innate mode, as well as not be efficient in translation. In addition, the high storage cost of the cold chain may limit its global availability and distribution. So improving RNA stability, intracellular delivery efficiency, and storage conditions are currently a major area of research and development in RNA vaccines [5,7].

Moreover, bioavailability, effective delivery and duration of protective immune responses are still important issues for the further development and optimization of RNA-based vaccines [2,4].

### 1.2. Pharmaceutical Challenges Associated with RNA Instability

Although significant strides have been made in RNA vaccine design, considerable pharmaceutical issues still exist, with the unstable molecular structure being among the major barriers. RNA molecules are highly susceptible to degradation by ribonucleases that are widely found in biological environments. This rapid degradation can substantially shorten RNA half-life and reduce intracellular translation efficiency. RNA molecules are large, negatively charged macromolecules. As a result, their transport across cellular membranes is highly challenging. Effective intracellular delivery therefore requires specialized delivery systems. Aside from structural instability, environmental factors such as temperature variations, pH changes, oxidative stress, and ionic composition can greatly modify the RNA structure and accelerate its degradation [9,10]. In addition, RNA molecules can trigger innate immune responses by recognition via pattern recognition receptors. Although some immune activation is beneficial, excessive activation can diminish translation efficiency and adversely affect antigen expression. As a consequence, RNA stability and immunogenicity control strategies are crucial for ensuring the successful development of RNA vaccines [11].

### 1.3. Need for Integrated Analytical Characterization and Stability Assessment

The worldwide swift advance of RNA-based vaccines has drawn attention to the importance of analytical characterization in order to guarantee product quality, safety and efficacy during development and manufacturing. Nevertheless, the intricate design and degradation susceptibility of mRNA are serious challenges and must be addressed by careful analysis and quality control approaches [2]. These structural pieces are involved in ribosome recruitment, translational efficiency and transcript stability. The 5′ cap and poly(A) tail can be integrated co-transcriptionally or post-transcriptionally during the in vitro transcription phase [12]. These stabilizers provide structural stability. However, mRNA molecules remain highly sensitive to ribonucleases and exhibit structural variability during biosynthesis, processing, and storage. Furthermore, IVT synthesis can lead to the appearance of impurities (e.g., incomplete transcripts, abortive products and dsRNA), which may compromise vaccine safety and efficacy. Due to the large, negatively charged phosphodiester backbone and the inherently single-strandedness of mRNA, it also has more dynamic secondary structures that render samples heterogeneous and analytical characterization more difficult [3,12].

As a result, several CQAs like RNA integrity, sequence identity, 5′ capping efficiency, poly(A) tail length and impurity profiles are of paramount concern as components are developed [12]. Because no one method can account for all CQAs, integrated analyses integrating multiple orthogonal methodologies are necessary to guarantee quality, stability and effectiveness of RNA-based vaccines during manufacturing and storage [7,8].

This review aims to provide a comprehensive overview of the pharmaceutical and analytical considerations associated with RNA-based vaccine formulations, with particular emphasis on RNA-LNP stability, CQAs, analytical characterization strategies, and stability assessment approaches. In addition, the review discusses current formulation optimization strategies and regulatory considerations relevant to the development, manufacturing, and long-term storage of RNA-based vaccines.

## 2. Overview of RNA Vaccine Platforms (mRNA, saRNA) from a Pharmaceutical Perspective

**Figure 1:**
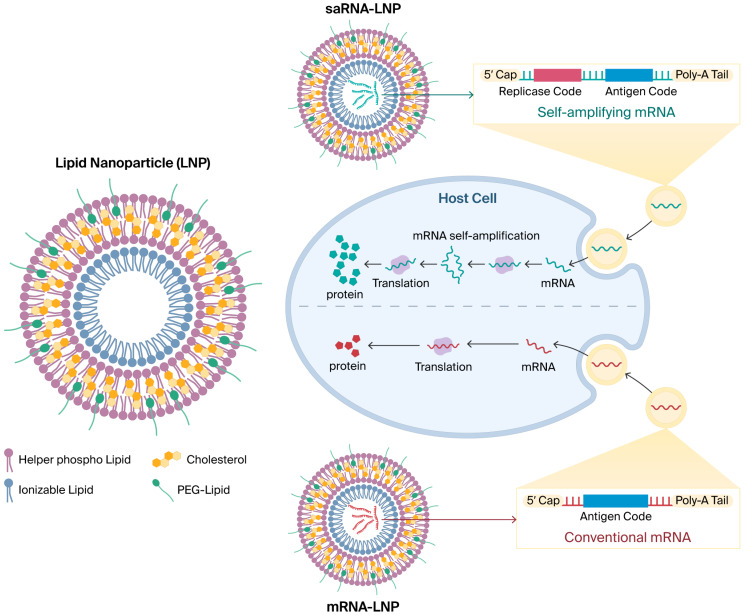
Schematic illustration of (RNA-LNP) structure and intracellular expression mechanisms of conventional RNA-LNP and saRNA-LNP vaccine platforms. RNA-LNPs are composed of ionizable ionic lipids, helper phospholipids, cholesterol, and polyethylene glycol (PEG) lipids. Conventional mRNA is schematically depicted as a sequence comprising a 5′ cap, a coding region that expresses the antigen, and a poly(A) tail. Upon entering the cell, the lipid nanoparticles are released into the cytoplasm, where they are directly translated into the target antigen. In contrast, saRNA contains an additional replicase-specific region, enabling intracellular amplification of the RNA prior to antigen translation. This diagram highlights the differences in delivery and intracellular expression mechanisms between conventional RNA-LNP and saRNA-LNP vaccine platforms. Created in BioRender. Alharthi, S. (2026) https://BioRender.com/uf9ivuz.

RNA vaccine platforms have revolutionized vaccine development globally, particularly for traditional mRNA and saRNA vaccines. These platforms have demonstrated unique pharmacological potential in terms of rapid manufacturing, formulation flexibility, and efficient immune response, as clearly demonstrated during the COVID-19 pandemic. The widespread clinical success of lipid nanoparticle-encapsulated mRNA vaccines highlighted the high potential of these platforms in terms of rapid development, flexible antigen design, and adaptability to viral variants [13]. Figure 1 shows the general structure of RNA-LNPs and the delivery mechanism of both conventional RNA-LNP and saRNA-LNP vaccines, highlighting the differences in their intracellular expression mechanisms. The success of mRNA vaccines depends not only on the antigen sequence but is also significantly influenced by formulation design, especially the type of ionizable lipids and the untranslated regions of the mRNA (UTRs), which are crucial for optimizing delivery, expression, and stability [13,14]. Despite the significant clinical successes of mRNA vaccines, several obstacles remain. These include the short duration of the immune response, the sensitivity of mRNA molecules to environmental conditions, and the relationship between physicochemical properties and functional efficacy remains poorly defined. This poses a significant challenge for the development of precise regulatory specifications for these products [15,16]. In this context, self-amplified/transcribed mRNA vaccines represent an advanced generation of mRNA vaccine platforms. These platforms are characterized by a different expression mechanism based on intracellular self-amplification followed by antigen expression, unlike the direct translation of traditional mRNA vaccines [15]. This self-amplification allows for longer antigen expression and a stronger immune response using lower doses (dose sparing) compared to conventional mRNA vaccines [17,18]. Preclinical studies have shown that saRNA-LNP vaccines are capable of generating strong and sustained humoral and cellular immune responses, while maintaining antibody levels for extended periods [17].

The use of saRNA-LNP vaccine platforms is not limited to combating the COVID-19 pandemic; it extends to other infectious diseases, such as respiratory syncytial virus (RSV). Preclinical studies have demonstrated that developing RNA-LNP vaccines that target specific antigenic regions leads to strong immune responses while maintaining a high level of safety, confirming the potential of these platforms in preventing a wide range of pathogens [19]. Overall, RNA vaccine platforms are promising technologies due to their rapid development, flexible design, and high immunogenicity. However, the continued success of these platforms depends on improving their formulation and deepening the understanding of the nonlinear relationship between the physicochemical properties of the formulation, its functional capacity, and the immune response. Quality indicators alone do not guarantee efficacy, which is a crucial foundation for developing vaccine platforms with broad and sustainable applications in the future [15,16]. For comparison, Table 1 presents a summary of the key differences between RNA-LNP and saRNA-LNP vaccine platforms from a pharmacological perspective, focusing on the mechanism of expression, RNA structure, dosage requirements, the sustainability of the immune response, and their relationship to the formulation’s properties.

### 2.1. Key Components of Lipid Nanoparticle Formulations and Their Functional Roles

RNA-LNP consists of four core components: an ionizable lipid, a helper phospholipid, a sterol such as cholesterol, and a polyethylene glycol (PEG)–lipid (PEG–lipid) [8]. The performance of RNA-LNP depends on ionizable lipids, which significantly influence their function. These lipids control apparent pKa, nucleic acid encapsulation, cellular uptake, and endosome escape efficiency, while helper phospholipids contribute to regulating membrane structure and rigidity [20]. PEG–lipid also plays a role in controlling particle size and preventing aggregation through steric repulsion [20]. This supports the quality of RNA-LNP and makes it more stable over time during storage, which serves pharmaceutical and vaccine applications [21]. PEG–lipid behavior may change in the biological environment when interacting with serum components; de-PEGylation occurs, promoting endosomal fusion, which is a crucial step for successful mRNA delivery [22]. Also, the changes in the PEG–lipid ratio affect the behavior of RNA-LNP. Higher PEG–lipid density and longer acyl chains may protect against aggregation and limit cell entry, thus prolonging blood circulation time. Conversely, lower PEG–lipid levels and shorter acyl chains may reduce stromal obstruction and improve cell entry [23,24]. When comparing the components of commercial vaccines to explain the difference in performance and storage stability, it was found that the ionizable lipid was the most influential component. SM-102 has been reported in some studies to exhibit improved performance compared to ALC-0315 [15].

Studies indicate that RNA-LNPs function not only as delivery systems but also as active modulators of immune responses. Ionizable lipids have been engineered by modifying the amino acid head so that it not only acts as a delivery component but also interacts with intracellular receptors to stimulate innate immunity [25]. Also, when a protein-based vaccine was administered with an empty RNA-LNP, an enhanced immune response was observed, indicating that RNA-LNP may act as immunomodulators even in the absence of mRNA [26]. Moreover, toxicity may vary depending on the RNA-LNP formulation and not solely due to mRNA or dose frequency [27]. Modifying the internal structure of lipids by altering the type of helper phospholipids and sterols may influence protein expression and immune response, indicating that their role is more than secondary [28]. As Blakney et al. have shown, the internal structure, particularly the helper phospholipid, plays a role in shaping the immunogenicity of vaccines [29].

### 2.2. Formulation Design Principles Influencing Vaccine Performance

Vaccine performance depends on the integration of RNA-LNP structure and mRNA molecule structure. The type of lipid used determines the behavior of the RNA-LNP, including its stability, interaction with the environment, cell entry, and endosome escape, while the structural properties of the mRNA affect its stability and translation efficiency [20,30]. Improving vaccines is not linked to greater protein expression or stronger activation of innate immunity, as this may lead to undesirable side effects. Rather, researchers should rely on modifying the RNA-LNP design by adjusting the properties of PEG–lipids, replacing cholesterol with plant sterols, or changing the type of helper phospholipids. This can reduce inflammatory responses while maintaining the strength of the immune response [31]. This is supported by comparisons between delivery platforms, which showed that the bioreducible polymer platform may cause higher levels of protein expression, while RNA-LNP may elicit a stronger immune response. These findings suggest that vaccine design should focus on the quality of the immune response, not just the level of protein expression [29]. From a structural RNA perspective, a study comparing the mRNA elements of two vaccines (Pfizer–BioNTec) and (Moderna) showed that improving vaccine potency may depend on the UTRs since they determine translation efficiency and mRNA stability [15]. In the context that RNA-LNP is not only a delivery tool, but it was also demonstrated in mouse models of multiply adjuvanted mRNA vaccines that both the carrier and the antigen itself participate in activating immunity, where an immunogenic ionizable lipid was used alongside an enhanced mRNA antigen. This may lead to an approximately tenfold increase in antibodies and allow for a reduction in the required dose [25].

### 2.3. Relationship Between Formulation Composition and CQAs

Critical quality characteristics in RNA-LNP platforms are not limited to the chemical composition and proportions of lipids, but extend to preparation conditions and the nature of the solutions used during the various manufacturing stages. Studies have shown that physical properties and biological performance can be affected by pH and the type and concentration of salts in different preparation solutions, such as mRNA solution, dilution, exchange, and storage solutions. PH is an important factor in improving packaging efficiency and cell expression, while the pH of the exchange solution contributes to biodistribution [32]. Post-processing strategies such as freeze-drying also have an impact on critical quality characteristics related to stability. The development of drying techniques has preserved particle size, packaging efficiency, and mRNA integrity, and freeze-dried vaccines have demonstrated a high degree of long-term stability [33,34]. In this context, the stability of RNA-LNP is affected by storage conditions, as refrigerated storage at −2 °C was the most stable for 150 days compared to storage at −20 °C. In addition, the use of trehalose and sucrose as protective materials contributed to reducing the agglomeration caused by freeze–thaw cycles and maintaining the stability and functional efficiency of the particles [35]. Achieving a strong immune response in RNA-LNP systems may be associated with safety challenges, especially with repeated doses, as it can lead to varying toxicity patterns. This highlights the importance of considering CQAs [27]. However, studies of stability under heat stress, through analysis of samples in terms of mRNA degradation, RNA-LNP size, and packaging efficiency, have shown that the relationship between these properties and functional capacity is not linear, governed by critical breakdown points; exceeding these points leads to a loss of effectiveness [16]. Packaging efficiency, calculated using common methods, and nanoparticle size are traditional quality indicators that may not accurately reflect the true performance of the formula. This underscores the need for indicators more closely related to functional behavior, both in vitro and in vivo [36].

## 3. Analytical Characterization of RNA-Based Vaccines

### 3.1. Morphological Analysis of RNA-LNPs

Cryo-transmission electron microscopy (Cryo-TEM) is the primary electron microscopy (EM) technique used to measure particle size and examine the morphology of RNA-based vaccine nanoparticles. However, it cannot directly measure the polydispersity index (PDI) or surface charge. These properties need extra methods, such as dynamic light scattering (DLS) for PDI and electrophoretic light scattering (ELS) for surface charge [37,38].

DLS reveals slightly larger sizes than cryo-TEM. For example, 91 nm versus 60–80 nm, and 80–85 nm versus 50–80 nm for PEG corona and hydration coatings, which are measured by DLS. These are invisible to cryo-TEM. Additionally, the intensity weighting inherent in DLS overemphasizes larger particles, which further increases the size discrepancy [39,40].

For instance, cryo-EM can detect small subgroups that DLS cannot detect. It can distinguish between subgroups with different structural subpopulations that contribute to the DLS peak [41]. The agreement between the charge detection mass spectrometry (CDMS) diameter distributions for empty RNA-LNP and the cryo-TEM [42] provides independent evidence that vitrification preserves particle dimensions.

From a morphological perspective, cholesterol analogs with C24 alkyl modifications induce polymorphic, faceted, and multilamellar morphologies [43,44]. The persistence of internal bilayer-like structures after pH neutralization is determined by the selection of an ionizable lipid [45]. Mixing methods, such as microfluidic mixing versus solvent injection, influence size and internal arrangement. Microfluidic mixing results in smaller, less-ordered particles, whereas solvent injection produces larger particles with highly ordered interlayer spacing of approximately 5 nm [46]. These morphological differences have functional implications. Faceted and multilamellar structures enhance gene transfection [44], while structures with a more homogeneous distribution of mRNA across subgroups (N/P = 6 vs. N/P = 3) exhibit higher transfection efficiency. The shift toward more effective morphological characterization and statistical accuracy has been enabled by the rise in automated image analysis tools and multimodal characterization platforms that combine asymmetrical-flow field-flow fractionation small-angle X-ray scattering (AF4-SAXS) with cryo-EM or convex lens induced confinement-alternating laser excitation (CLiC-ALEX) with cryo-TEM [37,38]. These developments address the fundamental limitations of low throughput and limited population sampling in cryo-EM analyses. Therefore, a detailed examination of individual particles is crucial for understanding the structure of RNA-LNP.

### 3.2. Analytical Evaluation of mRNA Encapsulation and Particle Homogeneity

Encapsulation efficiency (EE) and formulation homogeneity in RNA-based vaccines are evaluated using multiple analytical methods. However, complementary techniques are essential to ensure accurate and comprehensive characterization. RiboGreen is widely used to measure total encapsulation, while chromatographic techniques such as anion exchange chromatography (AEX), size exclusion chromatography (SEC), and ion-pair reverse-phase chromatography (IP-RP) to separate mRNA subsets and impurities. DLS and emerging single-particle techniques are also employed to uniquely identify the large proportion of empty RNA-LNP that are invisible to macroscopic analyses. The field is rapidly advancing, with ongoing improvements in analytical methodologies. The RiboGreen assay has become a reference method; however, additional techniques have been developed to overcome its known limitations [37,47,48].

Comparative studies highlight differences between RiboGreen and chromatographic separation methods, such as AEX, with a reported R2 = 0.67 across 30 samples. These discrepancies reflect not only measurement variability but also fundamental differences in detection principles. The RiboGreen assay measures the portion of mRNA that can bind a fluorescent dye, including free, surface-bound, and partially accessible mRNA. In contrast, AEX separates subsets chromatographically [49]. The two-dimensional liquid chromatography (2D-LC) workflow developed by [47] expands on this by providing simultaneous data on EE, integrity, adducts, and transcript ratio data [48]. EE values vary depending on the analytical method used. Agreement between methods is typically observed when the formulations contain minimal surface-associated mRNA. The differences between overall encapsulation efficiency measurements and single-particle analyses are notable. For instance, RiboGreen technology might indicate an EE over 90% even though it contains 40–80% empty RNA-LNP [50]. This is because most EE measures the total mass of encapsulated mRNA rather than the percentage of particles that are loaded. These two metrics reflect different quality aspects; mass-based encapsulation efficiency relates to the load, while the empty particle fraction pertains to the effective dose delivered by functionalized RNA-LNP. Conversely, single-particle techniques such as CLiC microscopy with ALEX, cylindrical illumination confocal spectroscopy (CICS), and nanoflow cytometry. These techniques enable the identification of population-level heterogeneity [37,45,50,51].

When evaluating particle size and homogeneity, choosing between DLS and Taylor Dispersion Analysis (TDA) produces different systematic results. TDA results for the intensity-weighted size distribution indicate a diameter of 86 nm for the same sample measured by DLS (128 nm), with a high PDI (0.011 vs. 0.112). These differences occur because DLS tends to overestimate larger particles due to intensity-based detection, while TDA provides a calibration-free measurement [52]. For polydisperse RNA-LNP samples, TDA might offer a more accurate analysis of the true size distribution. A comparison of SEC-MALS/DLS and Multi-detector AF4 (MD-AF4) shows that they provide more effective separation-based size determination than DLS, especially when large aggregates are present in small amounts [53,54,55].

The nature of the process for determining EE encapsulation efficiency and homogeneity is well-established. Continuous microfluidic mixing consistently outperforms batch methods in producing smaller, more monodisperse particles with higher EE [55]. Mixing flow rates, N/P ratio, and PEG–lipid ratio are among the most important formulation parameters and key processes [50,55,56]. Storage temperature and duration are the most critical post-processing factors that lead to formulation degradation. Methods such as Analytical ultracentrifugation (AUC), imaged capillary isoelectric focusing (iCIEF), and lipid formulation stability assay (LFSA) have been shown to indicate stability [57,58,59].

Evidence from previous studies indicates that no single analytical method is sufficient for comprehensive evaluation. Instead, a combination of orthogonal techniques is required. In conclusion, RiboGreen assays offer a simple preliminary measurement, while chromatographic methods (AEX, IP-RP, SEC, 2D-LC) provide high accuracy for identifying mRNA subsets and impurities. While single-particle techniques (CICS, CLiC-ALEX, and nanoflow cytometry) are essential for resolving heterogeneity at the group level, including a large proportion of empty RNA-LNP that conventional methods cannot detect, the trend is toward multi-attribute platforms that combine EE, integrity, size, and impurity assessment in a single analytical run (2D-LC [59], MD-AF4 [54]), emphasizing that these quality attributes are interrelated and preferably assessed together. Consequently, orthogonal characterization processes highlight the need for complementary methods rather than relying on a single technique [56,58]. Table 2 provides a summary of techniques.

### 3.3. Multi-Method Analytical Strategy for RNA Vaccine Quality Assurance

**Figure 2:**
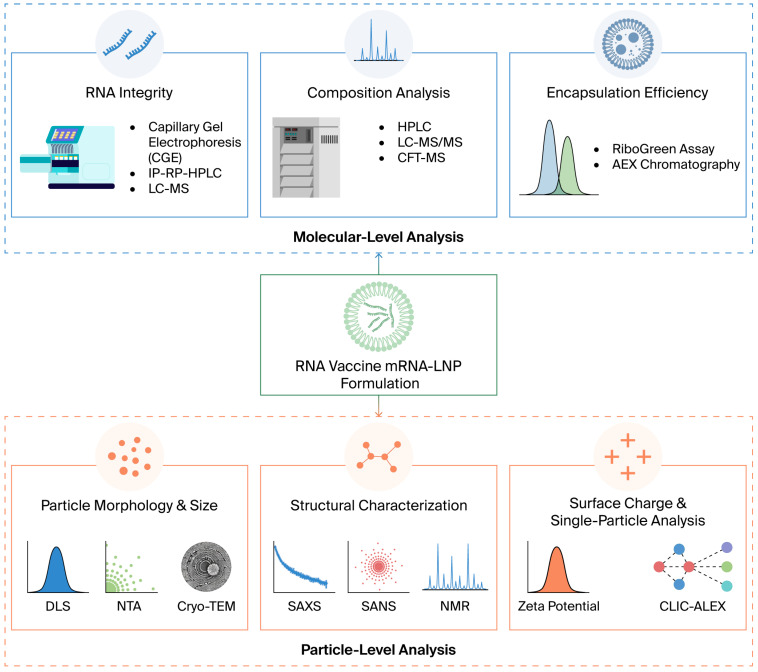
Overview of analytical techniques used to characterize RNA-LNP vaccine formulations. The diagram groups six complementary domains into two tiers. Molecular-level analyses assess RNA integrity (CE, IP-RPLC), composition (HPLC, LC-MS/MS, CFT-MS), and encapsulation efficiency (RiboGreen assay, AEX). Particle-level analyses assess morphology and size (DLS, NTA, cryo-TEM, CDMS, TDA), structural features (SAXS, SANS, NMR, MD-AF4), and surface charge at single-particle resolution (CICS, CLiC-ALEX). Together, these techniques address the key quality attributes required to assess the identity, integrity, and stability of RNA-based vaccines [37,38,41,45,46,47,53,56,57,58,60]; created by PowerPoint & figurelabs.

Analytical characterization plays a crucial role in ensuring the quality of RNA vaccines through a multi-method approach. Chromatography and mass spectrometry techniques verify mRNA sequence integrity and detect translationally silent lipid adduct products. Functional translation assays confirm protein expression and identify off-target frameshifts. Nanoparticle-level methods (nuclear magnetic resonance (NMR), scattering, and cryo-EM) address EE, RNA-LNP structural heterogeneity, and stability predictive attributes. No single method is sufficient to guarantee all these properties on its own, as illustrated in Figure 2. One of the key findings from the reviewed literature is that no single method is sufficient to guarantee the quality of RNA-based vaccine formulations. The quality attributes of RNA-LNP products vary across multiple levels, from nucleotide-level sequence fidelity to structural integrity at the nanoparticle level. Different methods exhibit both strengths and weaknesses [45,49,60,61].

Assessing mRNA integrity is a prime example of the complementary and interconnected nature of analytical and detection methods in ensuring the quality of RNA-based vaccine formulations. Although traditional CE methods are effective in detecting size-based degradation products, they fail to detect mRNA–lipid adduct products that render mRNA untranslatable [62,63]. IP-RP liquid chromatography methods (IP-RPLC or RP-IP HPLC) are effective in isolating and separating adducts from intact mRNA [48,54,61,62]. This means that CE-based integrity results may overestimate the proportion of functional RNA-LNP constructs. Similarly, unlike the RiboGreen fluorescence assay, which cannot differentiate among free, surface-bound, and fully encapsulated mRNA fractions. AEX provides high-resolution separation that differentiates these mRNA subpopulations. providing a more detailed view of encapsulation heterogeneity [49].

Overall, the literature supports a multi-tiered analytical strategy with three levels of analytical and detection methods.

First, there is chemical integrity (LC-MS/MS, IP-RPLC, and CGE), which assesses the integrity of the mRNA sequence and adducts at the molecular level [54,57,61,64]. Second, there are functional assays (CFT-MS) and cell-based transfection that confirm the correct translation of protein products [60,65]. Third, there are nanoparticle characterization methods (DLS, NMR, small-angle neutron scattering (SANS)/SAXS, cryo-EM, and AEX) that evaluate RNA-LNP structure, encapsulation, and surface properties [45,49,51,66]. Therefore, the appropriate combination of these methods depends on the development objective and quality considerations.

## 4. Structural Integrity and Purity Assessment

### 4.1. mRNA Structure: First Line of Defense Against Degradation

Since the 1990s, mRNA vaccine efficacy against influenza has been studied [67], leading to the most recent mRNA vaccine against COVID-19 [68], which has proven to be a transformative platform in vaccinology. mRNA is a precisely engineered molecule, optimized at multiple structural levels to resist degradation, evade off-target immunogenicity, and sustain translational output [69].

The importance of 5′ cap structure in eukaryotic mRNA translation efficiency has been well studied [70,71,72], yet it plays an important role in the safe degradation process without activating innate immune response [73,74]. The cap-0 structure (m7GpppN) is conserved across all eukaryotes and promotes translation initiation by facilitating eIF4E binding, while the cap-1 structure, which adds 2′-O-methylation to the first transcribed nucleotide, is the predominant form in higher eukaryotes and serves as a molecular self-identity signal that prevents innate immune recognition [75]. The predominant cytoplasmic mRNA decay pathway proceeds in the 5′-to-3′ direction, initiated by removal of the m7G cap by the DCP1/DCP2 decapping complex, which exposes the transcript to rapid 5′-to-3′ exonucleolytic degradation by XRN1 [76]. Cap-1 mRNA simultaneously resists degradation and evades innate immune recognition by marking the transcript as self [73,77].

Standard cap analogs can be incorporated into IVT transcripts in either orientation, generating a mixed population of functional and non-functional capped species that reduces overall translational yield by up to 50%. ARCA modification prevents reverse incorporation by blocking the 3′-O position of m7G, ensuring near-complete correct orientation and improving translational efficiency [78]. Vaccinia virus capping enzyme offers an alternative co-transcriptional approach with near-quantitative capping efficiency, as incomplete capping generates translationally inert and immunostimulatory uncapped species that compromise both expression and safety profiles of therapeutic mRNA [79].

#### 4.1.1. Untranslated Regions: Structural Buffers Against Decay

The 5′ and 3′ UTRs of an mRNA transcript are not passive flanking sequences. They are regulatory elements that interact with RNA-binding proteins, influence ribosome recruitment efficiency, and contain structural features that modulate both translation initiation and transcript stability [80].

The 5′ UTR harbors the consensus Kozak sequence immediately upstream of the initiator codon AUG, which governs ribosomal scanning efficiency [80,81]. A strong Kozak consensus (GCCRCCAUGG) reduces ribosomal stalling, which otherwise exposes the transcript to co-translational decay [82]. The stem-loop secondary structure in the 5′ UTR adds protection against degradation. Indeed, the S-loop structure stalls the scanning process of 40S near the initiation codon, adding more stable scanning of the AUG codon [82,83].

AU-rich elements (AREs) within the 3′ UTR serve as docking sites for destabilizing RNA-binding proteins such as TTP and AUF1, which recruit the CCR4-NOT deadenylase complex to accelerate transcript turnover [84]. Therapeutic mRNA constructs are therefore designed to exclude ARE-containing sequences, and instead incorporate 3′ UTR elements derived from highly stable endogenous transcripts such as the β-globin 3′ UTR, and combined AES/mtRNR1 sequences represent well-characterized examples that extend cytoplasmic transcript half-life and enhance protein expression [85,86].

#### 4.1.2. Modified Nucleosides: Silencing the Innate Immune Alarm

Unmodified IVT mRNA is recognized as foreign by innate immune sensors, including endosomal TLR7/8 and cytosolic MDA5/RIG-I, triggering type I interferon production and translational suppression that undermines antigen expression [87]. Nucleoside modification, initially demonstrated with pseudouridine (Ψ), was shown to suppress TLR7/8 activation and reduce MDA5-mediated signaling, allowing the modified transcript to bypass innate immune detection and undergo efficient translation [88]. Licensed mRNA vaccines subsequently adopted N1-methylpseudouridine (m1Ψ), which, beyond immune evasion, alters RNA base-stacking and hydrogen bonding geometry, modifying the secondary structure landscape of the transcript and reducing immunogenic dsRNA byproduct formation during IVT [89].

#### 4.1.3. The Poly-A Tail: Synergy at the 3′ Terminus

The 5′ cap and poly(A) tail function synergistically to enhance translational efficiency, with neither element alone accounting for the full stimulatory effect observed when both are present [70]. This synergy is mediated by PABP, which simultaneously binds the poly(A) tail and interacts via eIF4G with the cap-bound eIF4E, forming a closed-loop mRNA configuration [90] that stabilizes the translation initiation complex and protects the transcript from degradation [91].

Poly(A) tail length has a nonlinear relationship with translational efficiency, with an optimal length of approximately 75–100 nucleotides supporting maximal PABP binding and closed-loop formation, while further extension beyond 100 nt confers diminishing translational benefit [92]. Therapeutic mRNA vaccines typically employ poly(A) tails of 100–150 adenosine residues, which represent the practical optimum for stability and expression [93,94]. Template-encoded tails provide sequence uniformity, whereas enzymatic post-transcriptional addition generates length heterogeneity that is analytically detectable and constitutes a critical quality attribute requiring characterization [85]. The structural integrity of these mRNA elements is therefore a critical quality attribute requiring robust analytical characterization throughout development and release testing [3].

#### 4.1.4. From Structure to Function: Integrity–Performance Relationships

Degraded mRNA integrity produces measurable deficits in translational performance and immunogenic responses, creating a fundamental connection between molecular quality characteristics and vaccine therapeutic outcomes [3]. Compromised cap structures significantly impair translation initiation efficiency, as cap-dependent recognition machinery requires intact cap-binding proteins like eIF4E to effectively recruit ribosomal complexes to mRNA [72]. Studies demonstrate that cap1 structures, featuring additional 2′-O-methylation, exhibit enhanced translational performance and superior resistance to innate immune recognition compared to cap0 variants, with cap1 modifications providing better protection against degradation and improved translation efficiency [74].

Poly(A)-binding protein (PABPC) requires approximately 27 nucleotides for optimal binding, corresponding to its molecular footprint, though cap-dependent translation remains largely independent of poly(A) tail length within the 5–50 nucleotide range [92]. Research has identified 75 nucleotides as a critical threshold length, with this specific tail length promoting formation of a double closed-loop mRNA structure that enhances translation efficiency beyond typical cap-poly(A) synergy [92]. The general principle of cap-poly(A) cooperative function supports enhanced translational control, with both structural elements working synergistically to optimize gene expression [70,90]. Double-stranded RNA is an inherent byproduct of in vitro transcription, arising from the aberrant activity of T7 RNA polymerase and constituting the principal contaminant in IVT-derived mRNA preparations [95]. Once intracellularly delivered, these dsRNA byproducts are sensed by multiple pattern recognition receptors, including the cytosolic sensors RIG-I and MDA5, endosomal TLR3, the translational regulator PKR, and the 2′,5′-oligoadenylate synthetase (OAS) pathway, collectively triggering type I interferon production, global arrest of protein synthesis, RNA degradation, and in some cases cell death, all of which detrimentally impact mRNA therapy efficacy [96]. At the molecular level, PKR phosphorylates eukaryotic initiation factor eIF-2α to block translational initiation, while OAS activates RNase L to globally degrade cellular RNA, both representing distinct yet converging mechanisms of translational shutdown [97,98]. The immunological consequences of IVT-derived dsRNA are nonetheless not uniformly detrimental; they operate as a double-edged sword whereby low dsRNA concentrations may serve as endogenous adjuvants that support innate immune priming, while excessive levels markedly suppress antigen expression and adaptive immune responses in a manner that varies according to antigen sequence composition and structural properties [99]. Optimized IP-RP-HPLC chromatographic purification effectively removes dsRNA and hybridized RNA fragments from IVT preparations, substantially reducing innate immune activation while improving translational output [100]. These multifaceted consequences underscore the critical importance of systematically assessing and controlling dsRNA levels throughout IVT manufacturing as a determinant of both safety and therapeutic efficacy [101].

### 4.2. RNA Integrity Assessment

RIN was developed and validated on approximately 1300 total RNA samples from human, mouse, and rat tissues, with 28S peak height and the 18S/28S ribosomal ratio serving as its two primary algorithmic features [102]. Single-species therapeutic mRNA preparations produce a single dominant electrophoretic peak without these ribosomal landmarks, placing them outside the validation domain of the RIN algorithm. CGE and microfluidic electrophoresis platforms are therefore the preferred tools for mRNA integrity assessment, separating full-length transcripts from truncated and degraded species to yield a percent full-length metric [103,104]. Both platforms separate molecules by size and cannot distinguish capped from uncapped transcripts, which co-migrate under denaturing conditions; cap identity requires orthogonal LC-MS analysis [103,104]. A further limitation is that mRNA higher-order structure generates artifactual peaks in both CGE and LC chromatograms, producing anomalous integrity readouts that are difficult to correlate with bioinformatically predicted structural conformations [105]. Conventional CGE separations require 40–60 min per sample, which restricts throughput in routine quality control settings [103]. Microchip CE reduces this to approximately 70 s per sample, enabling a full 96-well plate in approximately 2.5 h at comparable resolution [3]. CGE can additionally determine average poly(A) tail length following RNase T1 digestion and magnetic oligo(dT) bead purification, enabling simultaneous integrity and tail-length assessment on a single platform [64].

Nanopore sequencing further extends this multiattribute capacity through the VAX-seq workflow, which provides PCR-free, single-molecule characterization of transcript integrity, poly(A) tail length, purity, and nucleoside modifications in a single experiment; cDNA-based long-read sequencing is the preferred approach for modified mRNAs, as direct RNA sequencing produces systematic errors at N1-methylpseudouridine positions [106]. However, standard nanopore protocols cannot access the true 5′ terminus of RNA molecules, limiting cap structure characterization [107]. TERA-Seq addresses this by ligating sequence-specific adapters to both molecular termini, enabling true end-to-end, single-molecule native RNA sequencing with precise resolution of both capped and non-polyadenylated species [107].

**Figure 3:**
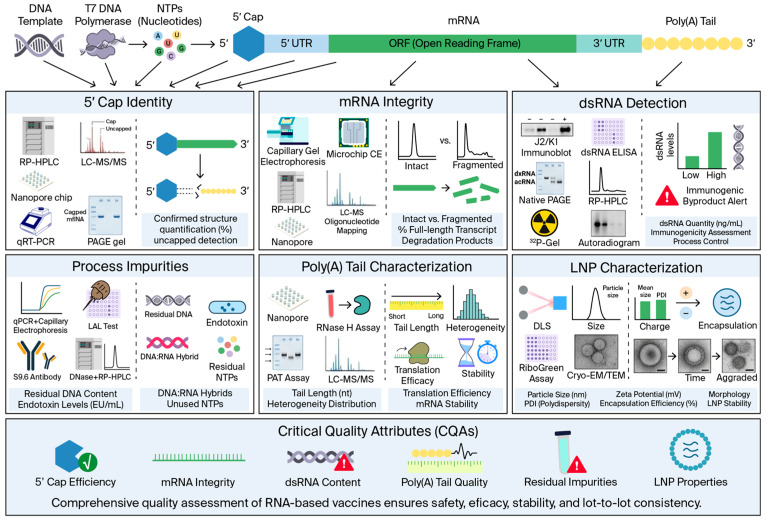
Comprehensive analytical strategies and critical quality attributes (CQAs) for quality assessment of IVT-derived RNA vaccine drug substances (adapted from [108]). The figure summarizes six major analytical domains used to evaluate RNA vaccine quality. 5′ cap identity is assessed using RP-HPLC, LC-MS/MS, nanopore sequencing, qRT-PCR, and gel electrophoresis to confirm cap structure, capping efficiency, and uncapped transcript content. mRNA integrity is evaluated using capillary gel electrophoresis (CGE), microchip CE, RP-HPLC, LC-MS oligonucleotide mapping, and nanopore sequencing to determine full-length transcript integrity and degradation profiles. dsRNA byproducts are detected using immunological and chromatographic approaches, including J2/K1 immunoblotting, dsRNA-specific ELISA, native PAGE, RP-HPLC, and radiolabeled gel electrophoresis. Process-related impurities are monitored through assays for residual DNA, endotoxin, RNA hybrids, and residual nucleotides. Poly(A) tail characterization is performed using nanopore sequencing, RNase H assays, PAT assays, and LC-MS/MS to assess tail length, heterogeneity, translational efficiency, and RNA stability. Lipid nanoparticle (LNP) characterization includes particle size, polydispersity index (PDI), zeta potential, encapsulation efficiency, morphology, and formulation stability using DLS, RiboGreen assays, and cryo-EM/TEM. Collectively, these analytical approaches define the critical quality attributes (CQAs) of RNA-based vaccines, including cap efficiency, mRNA integrity, dsRNA content, poly(A) tail quality, residual impurities, and LNP properties, ensuring product safety, efficacy, stability, and lot-to-lot consistency. Created by Figurelabs.

Therapeutic mRNA preparations contain several classes of process-related impurities, including fragmentation products, dsRNA byproducts, abortive transcripts, uncapped species, and poly(A) tail variants, each requiring a distinct analytical strategy (Figure 3) [3]. Fragmentation products and truncated species are primarily resolved by CGE and IP-RPLC, which separate them from full-length transcripts based on size and hydrophobicity, respectively, though species closely approximating full-length transcript size remain below standard electrophoretic resolution thresholds [103]. Reversed-phase HPLC is the long-standing downstream approach for removing dsRNA from IVT products. Its complexity and cost were the principal drivers behind efforts to engineer a low-dsRNA T7 RNA polymerase variant [108,109]. Cellulose-based chromatography has since emerged as a practical and lower-cost alternative. It exploits the preferential adsorption of dsRNA to cellulose fibers in a 16% ethanol buffer, achieving removal of at least 90% of dsRNA contaminants with good mRNA recovery [95]. For analytical monitoring, the J2 monoclonal antibody immunodot blot is the most widely adopted method. It detects dsRNA species of 40 bp and above, though species shorter than this threshold fall outside its detection window [95,98]. Where a quantitative readout is needed for quality control, dsRNA-specific ELISA offers the sensitivity and throughput required in a manufacturing setting [98].

The 5′ cap and 3′ poly(A) tail are terminus-level CQAs whose characterization requires prior nuclease digestion to generate terminus fragments suitable for chromatographic and mass spectrometric analysis [3]. Capping efficiency is assessed by LC-MS following RNase H- or DNAzyme-mediated cleavage of the 5′ terminus. Capped and uncapped species are distinguished by their distinct masses in extracted ion chromatograms and deconvoluted mass spectra [3]. Poly(A) tail heterogeneity arising from incomplete enzymatic addition or partial hydrolysis during storage is characterized by IP-RPLC, which resolves individual length variants up to 150 nt [3]. SEC provides a simpler and more robust complementary measure of average tail length, with LC-MS serving as the confirmatory orthogonal method [3]. Because IP-RPLC is more complex and less robust than SEC, the two approaches are typically deployed in combination. CGE additionally offers superior resolution of pre-peak impurity species, particularly in stability studies where tail degradation products accumulate [3].

## 5. Potency and Bioassays

### 5.1. In Vitro Translation Assays for Functional Evaluation

CFT technology allows fast and simplified sample preparation and prototyping for protein translation, without the negative impacts of RNA-LNP. Cell-based translation (CBT), on the other hand, provides a full understanding of mRNA functionality in vivo [61]. As summarized in Table 3, translation-based potency assays offer distinct advantages depending on the application. Although there are several translation methods with unique applications in this field, detecting translated proteins using antibodies remains challenging. These limitations were investigated by Stiving et al. by developing a new antibody-free system using LC-MS/MS, providing more accurate protein detection via CFT or CBT inputs, making it suitable for rapidly evolving viruses [61].

Functional analysis of circular RNA (circRNA) is conducted following the identification of their location and the mechanism of action; the goal is to elucidate their roles in cellular physiology via two major mechanisms: gain-of-function and loss-of-function approaches [110]. Overexpression studies using circRNA expression plasmids, transposon systems, or viral vectors to assess their effects on cellular proliferation, chemoresistance, and autophagy. Loss-of-function analysis approaches like siRNA-mediated targeting of the back-splice junction (BSJ) probe for targeted shRNA-mediated stable silencing by different mechanisms, including circRNA degradation and viral delivery, and circRNA degradation genome editing techniques, such as CRISPR-Cas9 and CRISPR-Cas13, precisely disrupt circRNA biogenesis and cleave target circRNA transcripts, respectively. The function of the modulated circRNA is then assessed by several methodologies to test the following: cell viability, proliferation, tumorigenic potential, and apoptosis, thus offering a comprehensive characterization of circRNA roles in both health and disease contexts [110,111].

### 5.2. Cell-Based Potency Assays and Their Relevance

**Figure 4:**
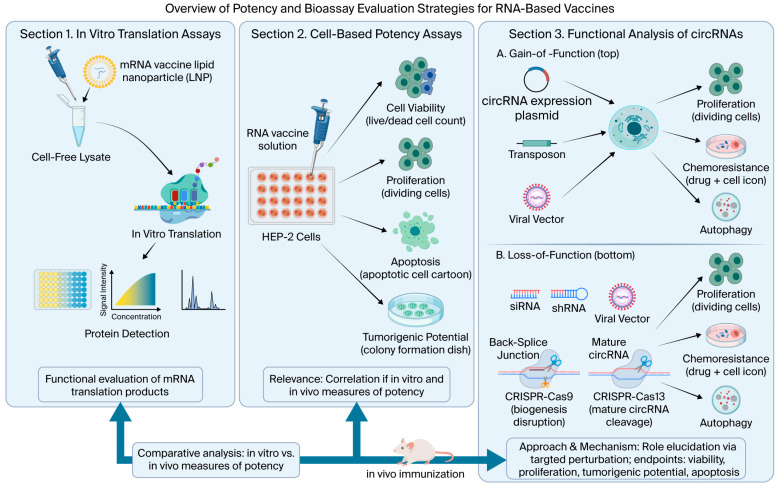
Overview of potency and bioassay evaluation strategies for RNA-based vaccines. (**Section 1**) displays an in vitro translation approach for functional evaluation. While (**Section 2**) illustrates the cell-based potency strategy for a candidate RNA vaccine. (**Section 3**) shows the functional analysis of circRNA, and the gain-of-function (**A**) and loss-of-function analysis approaches (**B**). Created by Figurelabs.

Synthesizing potent RNA vaccines is crucial for vaccine and drug development and for the release of clinical drug product lots and stability evaluation. Although animal studies can provide insight into the potency of targeted vaccines by demonstrating technical alignment with the hypothesized mode of action of the candidate vaccine, these studies are expensive, time-consuming, require skilled individuals to conduct the experiments, and necessitate the use of animals, which are not always available. Thus, developing an alternative in vitro approach was needed to overcome this limitation (Figure 4). Several studies have been completed or are still under investigation to compare in vitro and in vivo measures of potency [112]. For instance, Feller et al. compared the efficacy of in vitro assays with the in vivo immunization strategy using the BALB/c model. Notably, statistical analysis showed that the Dose 50 s (ED50s) from the in vivo study correlated with cell-based relative potency. This suggests that a cell-based approach may replace animal immunogenicity studies for mRNA-based vaccines and address their associated limitations [113].

### 5.3. Influence of Formulation on Biological Activity

mRNA-based vaccines are usually designed by cell-free transcription and then injected with a targeted delivery system to overcome the obstacles associated with crossing cellular membrane barriers. Therefore, different formulation alternatives have been developed for more sufficient cytosolic release, such as electroporation, RNA-LNP, protamine complex, and cell-based delivery systems. The protamine-based approach facilitates the uptake of mRNA by using electrostatic interaction, which activates TLR7/8 signaling and induces a robust immune response, indicating a potential strategy for preventing and treating infectious diseases and cancer, even though it does not remain in circulation for long periods [114,115]. Furthermore, RNA-LNPs are widely used as a delivery system, due to their ability to keep the desired mRNA intact and protect it from degradation, provide high-rate cellular uptake and facilitate endosomal escape (Figure 4).

Optimized lipid compositions improve the induction of both strong arms of the immune responses, cellular and humoral immune response, by enhancing antigen expression; it is also considered safe, supporting its usage in cancer immunotherapy and the vaccine field in general. Furthermore, electroporation and dendritic cell (DC)-based delivery systems enhance antigen presentation and T-cell activation and proliferation, especially in cancer immunotherapy. Collectively, optimizing mRNA formulations is critical for successful RNA therapeutics and vaccine development [115].

### 5.4. Challenges in Standardizing Potency Testing for RNA Vaccines

The evaluation of the quality, safety, and efficacy of RNA-based vaccines for treating diseases such as cancer and infectious diseases is now being regulated. The challenge now is to establish sufficient manufacturing protocols for more efficient synthesizing and delivering RNA-based vaccines for preventive and therapeutic purposes [30]. Specifications for manufacturing process acceptance criteria and materials used during drug synthesis and drug product should be well defined, e.g., with respect to manufacturing yields and the implementation of innovative analytical methods that provide specific quantification and thorough characterization of the product, including its identity, purity, and overall quality [30,116]. The quality of mRNA vaccines can be assessed using different well-established approaches, such as gel electrophoresis and HPLC, while strategies like sequencing techniques, which include next-generation sequencing and reverse transcription polymerase chain reaction (RT-PCR), are used for evaluating the identity [116]. Enzymes and solvents, any DNA residuals, the presence of endotoxins, truncated RNA fragments, and overall sterility, and the stability of the RNA vaccine should all be considered.

## 6. Formulation-Dependent Stability of RNA Vaccines

The success of mRNA vaccines represents a paradigm shift in immunology, yet their widespread deployment is controlled by a critical limitation: the inherent instability of the RNA molecule. Naked mRNA is highly susceptible to rapid degradation by extracellular RNases and chemical hydrolysis. Therefore, formulation strategies are essential for preserving vaccine potency [9,117]. Currently, RNA-LNPs serve as the primary delivery vehicles, encapsulating the mRNA to shield the genetic payload and facilitate cellular uptake. The precise ratio of ionizable lipids, cholesterol, and PEGylated lipids determines the thermodynamic stability of the complex [118]. The chemical composition dictates the vaccine’s sensitivity to temperature, directly influencing shelf-life and the rigorous “cold chain” logistics required for distribution [119,120]. Consequently, optimizing formulation is not just about efficacy, but about engineering thermal resilience to ensure global accessibility.

### 6.1. Chemical and Physical Degradation Pathways of RNA

**Figure 5:**
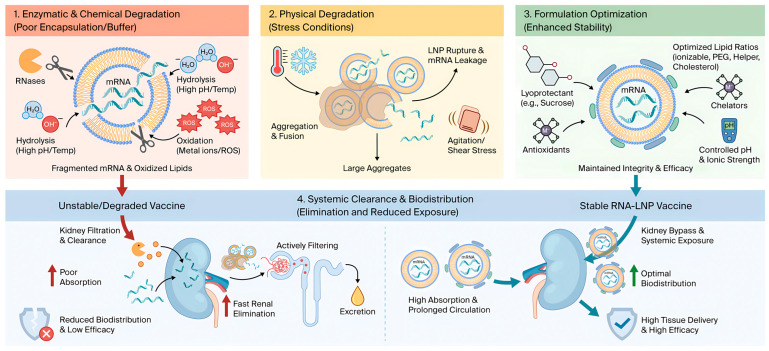
The schematic illustrates the major mechanisms responsible for mRNA-LNP instability and clearance, as well as formulation strategies used to enhance vaccine performance. (**1**) Enzymatic and chemical degradation: Poor encapsulation or unsuitable buffer conditions expose mRNA and lipids to RNases, hydrolysis, oxidation, ROS, and metal ions, causing mRNA fragmentation and lipid oxidation. (**2**) Physical degradation: Environmental and mechanical stressors, such as temperature fluctuations, freeze–thaw cycles, agitation, and shear stress, can induce lipid nanoparticle aggregation, fusion, structural damage, and mRNA leakage, reducing formulation stability. (**3**) Formulation optimization: Stabilizing excipients and optimized lipid compositions, including ionizable and helper lipids, cholesterol, PEG–lipids, antioxidants, chelating agents, lyoprotectants, and controlled pH/ionic conditions, help preserve nanoparticle integrity and maintain mRNA functionality. (**4**) Systemic clearance and biodistribution: Unstable or degraded RNA-LNPs are rapidly cleared by the kidneys, resulting in poor absorption, limited tissue distribution, and reduced efficacy. In contrast, stable, optimized RNA-LNPs exhibit prolonged circulation, improved biodistribution, enhanced tissue delivery, and greater biological activity. Created in BioRender. (2026). https://BioRender.com. Beyond chemical instability, physical degradation compromises RNA’s structural and functional integrity through environmental energy or mechanical stress. For instance, elevated temperatures lead to thermal fragmentation by increasing the molecule’s kinetic energy. This accelerates the shift toward “random coil” transitions, exposing the backbone to hydrolysis and causing significant fragmentation if RNA is stored for prolonged periods above −80 °C [9,125]. Mechanical stress can also physically break the molecules; the shearing forces caused by ice crystal formation during repeated freeze–thaw cycles easily damage long RNA strands. Furthermore, environmental radiation, particularly exposure to ultraviolet light, triggers photochemical reactions that result in base modifications or cross-linking, fundamentally altering the physical structure of the RNA [9]. Finally, physical stability is heavily influenced by structural remodeling. Intact secondary structures, such as hairpins and stem loops, effectively shield the RNA backbone from physical and chemical disruptions. Conversely, denaturing conditions driven by high heat or reduced ionic strength unfold these protective structures, greatly increasing the molecule’s vulnerability to degradation [9].

RNA degradation is driven by multiple mechanisms that depend on the molecule’s inherent chemical vulnerabilities and various physical factors (Figure 5). A thorough understanding of these degradation pathways is crucial not only for maintaining RNA homeostasis within living organisms but also for ensuring the stability of RNA-based therapeutics outside the body (ex vivo) [9,121].

The fundamental chemical instability of RNA stems from the 2′-hydroxyl (2′-OH) group on its ribose ring, which makes its phosphodiester backbone much more susceptible to hydrolysis than that of DNA. Specifically, this hydroxyl group acts as a nucleophile that attacks the adjacent phosphorus group, forming a 2′,3′-cyclic phosphate intermediate that ultimately breaks the RNA strand [9]. Because this chemical degradation relies on hydrolytic cleavage, the entire process is highly sensitive to the pH of the surrounding environment. In alkaline conditions, the deprotonation of the 2′-hydroxyl group actively drives self-cleavage [2], while at neutral pH, the presence of divalent metal ions such as Mg^2+^, Mn^2+^, Zn^2+^, and Ca^2+^ can catalyze this same reaction [9,122]. Moreover, RNA is susceptible to oxidative damage from reactive oxygen species (ROS) like hydroxyl radicals, which can alter the ribose sugar and nitrogenous bases to generate 8-oxoguanine, ultimately resulting in strand breaks or the premature termination of translation [9,123]. Within biological systems, chemical degradation is primarily controlled by enzymatic catalysis via ribonucleases (RNases). These enzymes actively exploit the 2′-OH group to hydrolyze phosphodiester bonds, with endonucleases (such as RNase E) cleaving the RNA internally, and exonucleases (such as Xrn1 and Dis3) degrading the molecule from its 5′ or 3′ ends [9,124].

### 6.2. Impact of Formulation Composition on Degradation Kinetics

The kinetic stability of mRNA vaccine is determined by the thermodynamic energy barrier required in initiating the cleavage of the phosphodiester bond and the oxidation of lipids [119]. Although the primary mechanism of mRNA degradation is an in-line nucleophilic attack by the 2’ hydroxyl of the neighboring phosphate group, the rate constant (k) of this process is influenced by the physicochemical environment provided by the RNA-LNP formulation [126]. Consequently, the formulation composition, specifically the ionizable lipid chemistry, buffer species, and cryoprotectants, acts as the primary determinant of the shelf-life and degradation kinetics of the drug product [119,120,126].

Chemical stability issues are associated with ionizable lipids, despite their importance. Recent kinetic studies on ionizable lipids containing tertiary amines suggest that N-oxide byproducts are formed, reacting with the mRNA backbone to produce covalent lipid–mRNA products that are translationally inactivated [127]. Additionally, the apparent $pK_{a}$ of ionizable lipids plays a crucial role in influencing intracellular pH; an acidic microenvironment within the RNA-LNP can accelerate acid-catalyzed hydrolysis of the mRNA phosphodiester bond, following pseudo-first-order kinetics. Advanced biodegradable lipids are now being engineered with specific ester linkages to optimize half-life, yet their hydrolysis kinetics must be balanced to prevent premature destabilization of the RNA-LNP structure during storage [118].

Beyond the lipid component, the aqueous phase composition plays a pivotal role in thermodynamic stabilization. The choice of buffer significantly impacts degradation rates; for instance, Tris buffers have been shown to mitigate ionizable lipid degradation more effectively than phosphate-buffered saline (PBS) by scavenging free radicals and maintaining a stable pH profile, thereby reducing the rate of oxidative degradation [32]. Furthermore, the inclusion of cryoprotectants such as sucrose is essential for modifying the glass transition temperature ($T_{g}$) of the lyophilized or frozen matrix. Maintaining the storage temperature well below $T_{g}$ restricts molecular mobility, thereby kinetically trapping the mRNA in a stable conformation and minimizing the frequency of collision-dependent degradation events [32,126].

### 6.3. Stability of RNA Versus Nanoparticle Structural Stability

The therapeutic efficacy of mRNA vaccines depends on the synergistic preservation of RNA’s covalent integrity and the design of colloidal nanoparticles. While RNA primarily confronts irreversible chemical hydrolysis, nanoparticles are susceptible to physical phase transitions and cargo leakage. Enhancing therapeutic vaccine efficacy by balancing RNA chemical and nanoparticle structural stability (Table 4).

### 6.4. Link Between Formulation Stability and Product Shelf-Life

The determination of product shelf-life is primarily dependent on the comprehensive assessment of product formulation stability. Shelf-life is defined as the period of time that a product will preserve its chemical, physical, microbiological, and toxicological specifications when kept under particular storage conditions [129]. Consequently, formulation stability is not only a quality attribute but the primary determinant of the product’s commercial viability and safety [130].

In terms of chemistry, the degradation of the active pharmaceutical ingredient (API) is the most direct regulator of the product’s shelf-life. Instability pathways such as hydrolysis, oxidation, and photolysis contribute to a loss of influence and the potential formation of toxic degradation products. The major chemical and physical degradation pathways affecting RNA vaccine formulations, together with formulation engineering strategies used to improve stability and extend shelf-life, are summarized in Figure 5 [131]. For instance, a product that does not sufficiently control pH may increase the rate of hydrolysis kinetics, drastically reducing the effective period of the product’s stability [132]. However, physical stability is equally significant. Although an API remains chemically stable, physical instabilities such as phase separation in emulsions, aggregation in proteins, or polymorph changes in solid dosage forms can render a product unusable [133]. These changes often alter dissolution rates and bioavailability, effectively ending the product’s shelf-life before chemical degradation occurs [134]. To determine this link quantitatively, regulatory guidelines such as the ICH Q1A recommend rigorous stability testing protocols [135]. Accelerated stability testing is conducted by the manufacturer to expose the formulation to elevated temperatures and humidity levels. The data obtained can be used to model the degradation rate using the Arrhenius equation ($K=Ae^{-Ea/RT}$) to calculate the shelf-life at ambient temperatures [136]. However, the most effective way to improve the formulation stability, thereby extending the shelf-life, is by optimizing the formulation variables [137].

## 7. Lipid Nanoparticle–RNA Interactions

The successful translation of mRNA therapeutics, particularly exemplified by the rapid development and deployment of COVID-19 mRNA vaccines, hinges critically on the intricate interactions between RNA cargo and its RNA-LNP delivery system [8,118,138]. These interactions dictate RNA protection from degradation, intracellular trafficking, and ultimately govern the functional performance and safety profile of the therapeutic product. A comprehensive understanding of the molecular and physicochemical forces at play is therefore paramount for the rational design and robust optimization of next-generation RNA-based nanomedicines.

### 7.1. Mechanisms of RNA Protection Within Lipid Nanoparticles

A primary function of RNA-LNP is to safeguard delicate mRNA molecules from premature degradation in the extracellular environment. Naked mRNA is inherently unstable and highly susceptible to enzymatic cleavage by ubiquitous ribonucleases (RNases), which drastically reduces its intracellular stability and subsequent translation efficiency [139]. RNA-LNPs provide a physical barrier, encapsulating the RNA within a protective lipid shell, thereby shielding it from hydrolysis and nuclease attack [8,140,141]. This encapsulation process is typically initiated under acidic conditions, where the positively charged amine headgroups of ionizable lipids electrostatically interact with the negatively charged phosphate backbone of mRNA [36,138,142]. Structural investigations, including those utilizing small-angle neutron scattering (SANS) and molecular dynamics (MD) simulations, have posited models wherein mRNA resides within an electron-dense core or within inverted micellar structures formed by ionizable lipids [141,143,144]. While one hypothesis suggests mRNA is situated within aqueous columns surrounded by cationic lipids, potentially exposing it to water and compromising stability under non-frozen conditions [145], MD simulations more broadly indicate that RNA entrapment is enhanced by the formation of internal water clusters, with RNA residing at the interface between these clusters and lipids [141]. This physical isolation and electrostatic complexation are foundational to preventing RNA degradation and maintaining its integrity throughout delivery. Critically, RNA-LNPs lacking encapsulated mRNA have demonstrated inferior stability compared to their mRNA-loaded counterparts, underscoring the cargo’s role in maintaining the overall particle robustness [145].

### 7.2. Role of Ionizable Lipids in RNA Complexation

Ionizable lipids are the cornerstone of RNA-LNP formulations, driving both mRNA complexation and subsequent intracellular release. Their unique pH-responsive nature is central to their function. At the low pH prevalent during RNA-LNP formulation, the amine headgroups of ionizable lipids become protonated, acquiring a positive charge that facilitates strong electrostatic interaction with the polyanionic mRNA. This charge–charge interaction is essential for efficiently encapsulating RNA-LNP [138,146]. A key quantitative parameter in this complexation is the nitrogen-to-phosphate (N:P) molar ratio, typically maintained around 6, which reflects the charge balance between the cationic lipid and anionic mRNA [138,141]. This ratio dictates the extent of mRNA complexation and influences RNA-LNP structural characteristics. Beyond simple charge interactions, molecular forces such as hydrogen bonding play a significant role. Ionizable lipids featuring hydroxyl groups can form additional hydrogen bonds with mRNA, thereby strengthening the lipid-mRNA binding affinity and minimizing mRNA loss during the LNP preparation process [142]. Furthermore, subtle variations in the linker region of ionizable lipids, specifically the number of methylene units between the headgroup and aliphatic tails, can modulate hydrogen bonding with the mRNA ribose-phosphate complex. This, in turn, influences the lipid’s apparent pKa and, consequently, its overall mRNA transfection potency [118]. These findings highlight that RNA complexation is not merely a bulk electrostatic phenomenon but a nuanced interplay of charge, conformation, and specific molecular interactions.

### 7.3. Influence of Molecular Interactions on Stability and Performance

**Figure 6:**
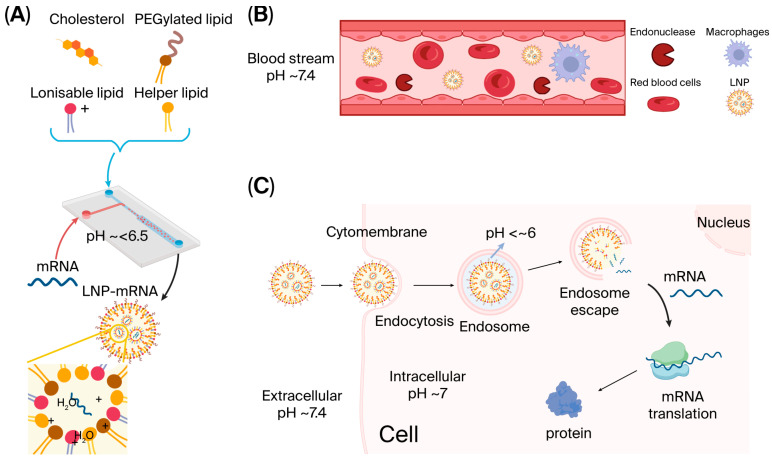
Mechanistic overview of lipid nanoparticle-mediated mRNA delivery from formulation to intracellular translation. (**A**) During formulation, protonated ionizable lipids electrostatically complex with negatively charged mRNA under acidic conditions, initiating RNA-LNP assembly. Within mature nanoparticles, mRNA is sequestered in lipid-rich domains that help protect it from enzymatic degradation. (**B**) Following systemic administration, RNA-LNPs remain largely neutral at physiological pH~7.4, improving colloidal stability during circulation. (**C**) After cellular uptake, endosomal acidification induces (pH~6) lipid reprotonation and membrane destabilization, enabling mRNA release into the cytosol, where it is translated to produce the encoded protein Created in BioRender. Altammami, M. (2026) https://BioRender.com/uwjbr9u.

The efficiency and safety of mRNA vaccines are profoundly influenced by the molecular and physicochemical interactions within RNA-LNP. The pH-dependent charge-switching characteristic of ionizable lipids is crucial for both RNA-LNP formation and mRNA release. At physiological pH (~7.4) in systemic circulation, these lipids remain predominantly neutral, minimizing non-specific interactions with anionic serum proteins and cell membranes, thereby reducing toxicity and preventing rapid clearance by immune cells [138,146,147]. However, upon cellular uptake, the acidic environment of endosomes (pH ~< 6.5) reprotonates the ionizable lipids, restoring their positive charge. This charge reversal triggers critical interactions with the negatively charged endosomal membrane, facilitating membrane disruption and the subsequent escape of mRNA into the cytosol for translation (Figure 6) [139,144,148]. This mechanism is widely accepted as a key determinant of RNA-LNP efficacy.

The apparent pKa of the RNA-LNP, a measure of the pH at which 50% of the ionizable lipid is protonated, is a critical physicochemical parameter influencing encapsulation efficiency, delivery efficacy, and toxicity [138]. Optimal apparent pKa ranges have been identified for different administration routes, with 6.2–6.6 for intravenous and 6.6–6.9 for intramuscular delivery, highlighting the need for tailored lipid design [138]. Discrepancies in pKa values between clinically approved vaccines, such as Moderna’s mRNA-1273 and Pfizer/BioNTech’s BNT162b2, underscore the complexity and the lack of a universal optimal pKa, suggesting other interacting factors are equally significant [149].

Beyond the headgroup, the molecular architecture of the ionizable lipid tails profoundly impacts RNA-LNP performance. Hydrophobic tails, whether saturated, unsaturated, or branched, influence crucial properties such as pKa, lipophilicity, membrane fluidity, and fusogenicity, all of which affect RNA-LNP formation and efficacy [138,149]. Branched-tail ionizable lipids, such as those used in ALC-0315 and SM-102 formulations, are thought to adopt a “cone” shape that promotes endosomal membrane instability and enhances intracytoplasmic release of nucleic acids [138,146]. Asymmetric tails in ionizable lipids have also been recognized for their ability to facilitate endosomal release, attributed to differences in their stretch and bending moduli compared to symmetric phospholipids [142]. Furthermore, MD simulations reveal that RNA concentration within the RNA-LNP can influence the molecular mobility of RNA-LNP components [150]. A lower mobility of ionizable lipids has been correlated with delayed protein production, suggesting that lipid dynamics play a crucial role in endosomal fusion and escape [150]. This interplay between molecular structure, dynamics, and the microenvironment determines the ultimate functional outcome of the RNA-LNP system.

### 7.4. Implications for Formulation Optimization

The implications of RNA-LNP interactions for formulation optimization and product robustness are extensive, demanding a multifaceted approach to design and characterization. The inherent chemical instability of lipids poses a significant challenge to long-term RNA-LNP storage. Oxidation of unsaturated hydrocarbon tails and hydrolysis of ester bonds within ionizable lipids can lead to the formation of dienone species, causing conformational changes, cargo degradation, colloidal instability, and ultimately loss of bioactivity [151]. To address this, buffer optimization strategies, such as the use of mildly acidic, histidine-containing formulations, have been shown to mitigate oxidative degradation and enhance room-temperature stability [151]. Particle size also plays a critical role in stability, with RNA-LNP in the 80–100 nm range exhibiting superior long-term stability of lipid components compared to larger particles [145].

For robust formulation, the careful optimization of the molar ratios of all four RNA-LNP components, ionizable lipids, helper phospholipids, cholesterol, and PEGylated lipids, is essential. These ratios significantly impact encapsulation efficiency, particle size, polydispersity, and therapeutic efficacy [138]. A higher total lipid concentration, for instance, has been shown to improve RNA capture efficiency [36].

From a characterization standpoint, reliance solely on traditional encapsulation efficiency (EE%) metrics can be misleading. Conventional EE% calculations often fail to account for RNA loss during the synthesis process, resulting in artificially high reported efficiencies. The implementation of an “input encapsulation efficiency” (EE_input%), which directly compares encapsulated RNA to the initial input RNA concentration, provides a more accurate assessment of reaction efficiency, enabling informed process optimization and reducing the waste of expensive RNA cargo [36]. Furthermore, overlooking the presence of intrinsic drug-free RNA-LNP within preparations can lead to underestimated core sizes and inaccurate mRNA loading quantification [142].

The rational design of ionizable lipids is crucial for enhancing overall RNA-LNP performance, including achieving tissue-specific delivery, efficient endosomal escape, and reduced toxicity [118,138,142,146]. The traditional, labor-intensive synthesis of these lipids can be expedited by modern approaches such as combinatorial chemistry and Passerini reactions, which facilitate the rapid generation of diverse lipid libraries for high-throughput screening [118,142]. Complementing these experimental strategies, artificial intelligence (AI)-driven rational design platforms are emerging, capable of predicting key RNA-LNP properties like apparent pKa and mRNA delivery efficiency, thereby accelerating the discovery of optimal ionizable lipid candidates [147]. Incorporating biodegradable chemical bonds, like ester linkages, into ionizable lipids is another critical strategy to minimize lipid accumulation and potential long-term toxicity, especially for therapies requiring repeat dosing [8,138,146]. Finally, the interactions between RNA-LNP and the innate immune system must be considered; ionizable lipids can elicit NF-kB and IRF responses through Toll-like receptor 4, representing a mechanism for innate immune activation that can be fine-tuned for adjuvant design and improved vaccine efficacy [152].

## 8. Role of Excipients and Manufacturing RNA Vaccine Processes

The recent literature increasingly emphasizes that the development of mRNA–lipid nanoparticle platforms is strongly influenced by formulation chemistry and manufacturing design [153]. While the mRNA sequence functions as the genetic blueprint for protein expression, multiple studies indicate that excipient composition and processing conditions are critical determinants of stability, safety, and large-scale accessibility of RNA-based therapeutics [154].

Excipients serve a fundamental stabilizing role in protecting the phosphodiester backbone of mRNA and maintaining the structural integrity of RNA-LNP systems during formulation and storage [121]. Previous research has shown that conventional phosphate-buffered saline (PBS) is often substituted with Tris or acetate buffers due to their improved physicochemical stability, particularly during lyophilization [155]. Tris buffers are frequently reported to reduce pH fluctuations associated with phosphate crystallization and to act as reactive scavengers that mitigate lipid-induced degradation, thereby enhancing mRNA stability [156]. Cryoprotectants such as sucrose and trehalose are widely recognized in the literature for their ability to form a protective glassy matrix around nanoparticles during drying processes, preventing aggregation and preserving encapsulation efficiency [157]. More recent investigations have explored polymer-based stabilizers, including polyvinylpyrrolidone (PVP), which improve moisture resistance and contribute to enhanced stability in solid-state RNA-LNP formulations [155]. In addition to excipient selection, the lipid composition of the four-component RNA-LNP system (ionizable lipids, helper lipids, cholesterol and PEG–lipids) has been consistently identified as a key factor governing delivery efficiency and endosomal escape. The molar ratio of these components directly influences particle size, encapsulation efficiency, and cellular uptake [158].

### 8.1. Effects of Formulation Processing and Mixing Methods

Manufacturing processes play an equally critical role in determining the final quality attributes of RNA-LNP products. The assembly of RNA-LNP is a process-dependent phenomenon in which nanoparticle morphology and size distribution are kinetically regulated by mixing dynamics [55]. Industrial formulation typically relies on microfluidic or T-junction mixing technologies, where rapid solvent exchange between lipid and aqueous phases enables controlled nanoparticle formation [153]. Key process parameters such as flow rate ratio (FRR) and total flow rate (TFR) significantly influence particle size distribution and PDI [55]. Post-formulation processing steps, including tangential flow filtration (TFF), introduce mechanical stresses such as shear forces that may affect nanoparticle integrity and encapsulation efficiency [116,156]. Furthermore, scale-up from laboratory to industrial production introduces challenges related to air entrainment, shear stress and surface-induced mRNA degradation, which can reduce encapsulation performance if not carefully controlled [157].

### 8.2. Manufacturing Scalability and Its Impact on Quality and Stability

Scalability remains a major bottleneck in mRNA vaccine manufacturing [116]. The transition from benchtop to industrial-scale production increases variability in purification, sterilization and thermal exposure, all of which may negatively impact mRNA stability and product consistency [153]. To overcome ultra-cold storage limitations, recent studies have focused on lyophilization, spray drying, and spray freeze drying [126,159] as alternative approaches to convert liquid RNA-LNP formulations into stable dry powders with extended shelf life under refrigerated conditions [160].

### 8.3. Relationship Between Manufacturing Parameters and Product Consistency

**Figure 7:**
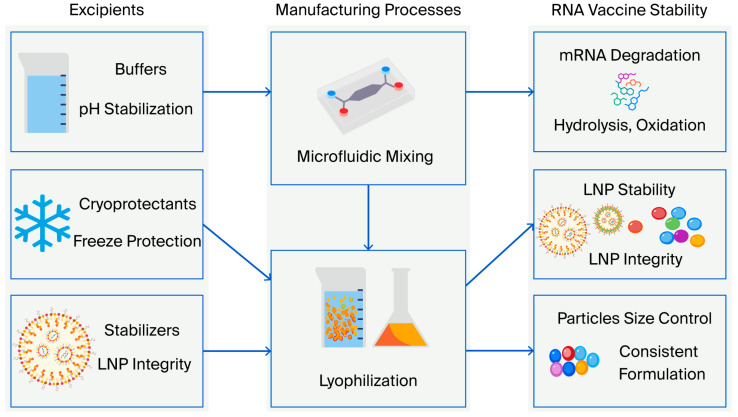
Impact of excipients and manufacturing processes on RNA vaccine stability. Excipients (buffers, cryoprotectants, and stabilizers) interact with key manufacturing processes such as microfluidic mixing and lyophilization, influencing CQAs including mRNA degradation, RNA-LNP stability and particle size distribution. Created in BioRender. alkhaldi, S. (2026) https://BioRender.com/aa3xpam.

The interaction between excipient selection and manufacturing process parameters represents a fundamental determinant of mRNA vaccine stability and performance. Previous studies have demonstrated that fluctuations in mixing energy, drying conditions, and purification processes can directly alter nanoparticle morphology, encapsulation efficiency and degradation kinetics. Therefore, a conceptual framework is required to illustrate how formulation variables and process engineering collectively regulate critical process parameters (CPPs) and CQAs throughout the production lifecycle [157]. Emerging approaches, including artificial intelligence (AI) and digital twin technologies, are increasingly being integrated into pharmaceutical manufacturing to enable real-time monitoring and predictive process control within a Quality-by-Design (QbD) framework [161,162]. Previous studies have highlighted the complex interplay between excipients, manufacturing processes, and the resulting stability of RNA-based vaccine formulations, as illustrated in Figure 7.

As summarized in Table 5, both formulation components and process-related factors act synergistically to determine the structural integrity and scalability of mRNA vaccine products across different manufacturing stages.

Analytical and Stability Evaluation of RNA-Based Vaccines: Characterization Strategies and Formulation-Related Challenges.

## 9. Regulatory and Analytical Frameworks

The introduction of mRNA vaccines has revolutionized the current mode of immunization [163,164,165,166], and the regulatory status of these vaccines remains somewhat hypothetical [167,168]. While they are legally classified as vaccines, their mode of action, with its nucleic acid component, remains the expression of proteins within the cell, and, with their use of nanoparticle delivery, they are essentially similar in their mode of action to gene therapy medicinal products [164,169]. Hence, the analytical/stability paradigm for mRNA vaccines remains more indicative of a level of regulatory control for gene therapies, with the requirement for harmonization and high resolution in the characterization [167,170,171].

### 9.1. Regulatory Expectations for Quality and Stability Assessment

In the United States of America, the Food and Drug Administration (FDA), through the Center for Biologics Evaluation and Research, controls mRNA vaccines [172]. In the European Union, the European Medicines Agency (EMA) controls mRNA vaccines [173]. The agencies use the International Council for Harmonization (ICH) guidelines on the quality of biotechnology-derived medicinal products [116,163]. Despite the fact that these vaccines fall under vaccine laws, they share common characteristics with gene therapies. They are able to present genetic information for the development of antigens instead of the administration of antigens [164,165]. This mode of intracellular delivery of genetic material mimics the mode of action of gene therapy medicinal products, even though the action of mRNA vaccines is transient and not genomic. The intricacy of the structural structure of the synthetic RNA-LNP delivery system demands standards of characterization similar to what is required for high-technology nucleic acid therapeutics [8,174]. Consequently, regulators increasingly require non-clinical studies and evaluations that mimic gene therapy assessments, while the large-scale production of these vaccines demands highly rigorous Chemistry, Manufacturing, and Controls (CMC) and quality assurance protocols [116,175].

### 9.2. Analytical Requirements Outlined by Major Regulatory Agencies

Moreover, the regulators require a lot of characterization of the drug product RNA-LNP and the drug substance (mRNA transcript) [121,168,171]. The CQAs include the following: sequence identity, 5′ cap integrity, poly(A) tail length, residual plasmid DNA, poly(A) contaminating impurities, encapsulation efficiency, particle size distribution, and potency [166,170,176].

### 9.3. Challenges in Regulating Emerging RNA-Based Platforms

An important regulatory problem concerns mRNA instability. The primary degradative pathways include hydrolysis and oxidation, which are mediated by the change in temperature [121,167]. The initial mRNA vaccines for the treatment of COVID-19 were required to be stored at −20 °C or −70 °C to preserve the molecular integrity, which may be exemplified as intrinsically labile [167,171]. Interestingly, the loss of stability is not only a structural phenomenon; translational inefficiency is mediated by RNA-LNP destabilization [145,174].

Hence, the assays that are likely to be used in the stability programs that are discussed in this article ought to include the stability-indicating assays that are capable of detecting the fragmentation of the RNA, the lipid peroxidation, and the potency loss [121,170,177]. When compared with traditional inactivated vaccines, where the antigen titer might be adequate, a functional cell-based expression assay is necessary for mRNA vaccines to prove biological activity [164,178]. The dependency of mRNA vaccines on functional potency is closer to regulatory expectations of gene therapy compared to traditional vaccinology [168].

Furthermore, for the mRNA vaccine, in silico models must be employed using orthogonal and precise approaches [121,166]. RNA-LNPs are defined as a class of nanoscale particles, typically in the range of 60–100 nm in diameter, composed of ionizable lipids, cholesterol, helper phospholipids, and PEG–lipids [174]. The size of the particles is a key feature that must be uniform in order to influence biodistribution and efficiency of uptake [145]. The encapsulation efficacy of approved COVID-19 vaccines is above 90%, which is essential because naked mRNA is highly susceptible to rapid enzymatic degradation in the extracellular environment [170,178].

The integrity of the RNA is determined by the use of electrophoresis and chromatography [121,166], while the particle size is determined by DLS [8,174]. Potency testing of the vaccine is carried out by translation analysis of the antigens, but not the presence of antigens [164]. The amount of impurities of dsRNAs should be reduced to a minimum, as it triggers the innate immune sensors and reduces the translation ability, hence affecting the performance of the vaccine [165,176,178].

### 9.4. Importance of Harmonizing Analytical and Formulation Strategies

Scalability of manufacturing processes is another factor that shapes analytical expectations. mRNA vaccine development processes can begin 2–3 weeks after identifying the sequence, a process considered faster compared to traditional manufacturing methods that require virus cultivation in embryonated chicken eggs [116,175]. However, this flexibility must be accompanied by confirmed comparability procedures, such that a change in processes does not affect CQAs [116]. The regularity of the platform is thus emphasized by the regulators, as well as analytical control measures [166,175]. Also, large synthetic mRNA products are new product types with unique issues for regulators [116,167]. Datasets for long-term stability are currently limited, although they are increasing, particularly in comparison to conventional vaccines [121,167,169].

Temperature sensitivity makes distribution logistics more complicated and makes it more reliant on cold-chain infrastructure [167,171]. There is also ambiguity of classification. The mRNA does not insert into the genome, but “conceptually, its therapeutic effect, that is, giving genetic instructions to produce endogenous protein synthesis, is similar to a form of gene therapy” [164,165]. It has been stressed that “nanoparticle-mediated delivery also raises nanomedicine-specific issues, e.g., lipid oxidation, and immunogenicity of PEG elements, which extend beyond traditional concepts of vaccines” [8,174]. Moreover, there is an “mRNA ‘platform’ nature, allowing rapid redesign as an antigen, but regulatory comparability in sequence changes is complex” [116,166]. It has been noted that successful scale-up requires a comprehensive understanding of critical quality attributes (CQAs) and robust analytical methods to demonstrate product comparability between manufacturing stages [174,175].

Additionally, there is a need to standardize globally as a means to ensure that the quality is assessed uniformly [116,171]. The stability of RNA, the integrity of RNA-LNP, and the efficiency of encapsulation are interdependent factors that influence immunogenicity [145,170,177]. The formulation of the drug and high-order analytics will provide the means to discover the degradation pathways and develop an intelligent mitigation plan [121,177]. The risks-based regulative science is becoming more common as the gene therapy-like nature of mRNA vaccination is complex with the use of nucleic acid molecules, vectors of delivery, and intracellular translation [168]. The analytical standards will provide the means to respond quickly to the pandemic while maintaining the high standard of quality [175]. Finally, mRNA products are controlled through a level of molecular and functional scrutiny at depths akin to those of gene therapy, despite their legal status as vaccines [164,165]. Their nucleic acid structure, fragility, and nanoparticle delivery platforms necessitate stringent, unified analytical principles in order to provide stability, uniformity, and global regulatory assurance [8,164,170].

**Figure 8:**
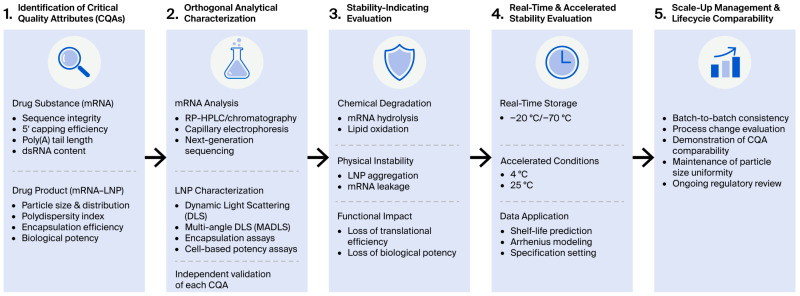
Regulatory framework for analytical and stability evaluation of RNA vaccine formulation. The figure illustrates a sequential, science- and risk-based CMC framework for RNA-LNP vaccine formulations. The model integrates identification of CQAs, orthogonal analytical characterization, stability-indicating evaluation of degradation pathways, real-time and accelerated stability testing for specification setting, and lifecycle management to ensure comparability during scale-up and manufacturing changes [8,116,121,145,165,166,167,168,170,171,174,175,177,178]. Created by PowerPoint & figurelabs.

The overall regulatory and analytical framework governing quality evaluation, stability assessment, and lifecycle management of RNA vaccine formulations is summarized in Figure 8.

## 10. Current Challenges

### 10.1. Intrinsic Instability of RNA and Sensitivity to Formulation Parameters

The phosphodiester backbone and nucleobases of RNA are prone to hydrolysis, oxidation, and nucleases, making it inherently unstable. The kinetics of degradation are extremely susceptible to handling stresses (agitation, freeze–thaw, and light) and microenvironmental variables (pH, buffer species/strength, ionic content, trace metals, and oxygen). According to [159,179,180], and others, stability in nanocarriers is linked to carrier integrity, lipid oxidation/hydrolysis, PEG–lipid loss, fusion/aggregation, and leakage, which can increase water exposure and accelerate fragmentation or cap/poly(A) damage. Even when bulk “integrity” measures barely change, lipid quality can also contribute to potency loss through reactive contaminants that chemically alter encapsulated RNA [181].

### 10.2. Limitations in Standardizing Analytical Methods Across Platforms

Standardization is limited by the range of CQAs related to RNA and nanocarriers, including RNA identity/truncation, capping and poly(A) heterogeneity, dsRNA/process impurities, as well as particle size distribution, shape, encapsulation, and lipid degradation products [3,12,182]. Non-equivalent sample preparation and extraction methods (detergents/solvents and denaturation conditions) may introduce bias in RNA readouts or compromise nanostructure, whilst orthogonal platforms (CGE/CE, LC methods, LC–MS, sequencing, DLS/NTA, cryo-EM) differ in sensitivity and feasibility for routine quality control [12,109]. Although guidance is emerging [183,184,185], reference standards and inter-laboratory comparability remain limited.

### 10.3. Complexity of Correlating Analytical Data with Long-Term Stability

Real-time potency and immunogenicity are not always predicted by analytical trends. Because several formulation-specific degradation processes might function concurrently, potency may deteriorate before noticeable changes in coarse physicochemical markers (such as mean size and encapsulation). RNA-lipid adducts and lipid oxidation products can lower expression without significantly altering bulk particle metrics or the electrophoretic full-length fraction [180,181]. Endosomal escape and translation efficiency may potentially be hampered by minute alterations in internal nanostructure. To map failure pathways and provide clinically meaningful acceptance criteria, prediction programs are increasingly combining orthogonal physicochemical assays with functional potency assays (cell-based expression) and targeted stress investigations [3,182].

### 10.4. Regulatory Challenges Associated with Emerging RNA Vaccine Technologies

Regulatory expectations are evolving, but experience is still dominated by first-generation RNA-LNP products. As modalities diversify (saRNA, circular RNA, and non-lipid nanocarriers), uncertainty persists around potency paradigms, comparability boundaries, and the evidence required for manufacturing changes, thermostable reformulations, and variant/antigen updates [183,184,186]. Platform-oriented tools (e.g., EU master-file concepts and FDA’s Platform Technology Designation) can accelerate follow-on products, but place greater weight on sensitive analytical comparability and risk-based bridging [187,188]. New Ph. Eur. general texts are also expected to tighten expectations for identity/purity controls and lifecycle management [185,189].

## 11. Future Perspectives

The rapid deployment of RNA vaccines has demonstrated the versatility and clinical potential of this platform, while simultaneously revealing a new set of scientific and technological challenges. Future progress will require an integrated strategy that combines advances in analytical characterization, digital and computational methodologies, and regulatory science to enable more stable, globally accessible, and clinically effective RNA-based vaccines.

The characterization of RNA vaccines is moving beyond conventional batch-release testing toward orthogonal, high-resolution approaches capable of probing product structure across multiple length scales. Techniques such as native mass spectrometry permit the analysis of intact RNA-LNP complexes, whereas cryo-electron tomography (cryo-ET) provides three-dimensional information on internal architecture under near-native conditions, including lamellarity, core density, and RNA distribution [190,191]. Emerging surface and depth profiling methods, notably cryogenic orbitrap secondary ion mass spectrometry (Cryo-OrbiSIMS), enable label-free mapping of the spatial distribution and molecular orientation of individual lipid species relative to the encapsulated RNA, thereby linking nanoscale organization to macroscopic stability and functional performance [37]. At the molecular level, next-generation sequencing (NGS) and microfluidic CE are increasingly important for confirming sequence fidelity, characterizing poly(A) tail length distributions, and detecting low-abundance impurities that may influence potency, reactogenicity, or innate immune activation [103].

These developments in analytical science are being coupled with real-time monitoring through broader implementation of Process Analytical Technology (PAT) [192]. In-line and on-line tools, including Raman spectroscopy, near-infrared spectroscopy, and DLS–based approaches, are being applied to monitor CQAs such as particle size distribution, turbidity, and encapsulation efficiency during microfluidic or impingement jet mixing. Real-time acquisition of such data allows rapid identification of deviations in critical process parameters (CPPs) and supports feedback control to maintain operation within a defined design space [193,194,195]. These data-rich environments are complemented by digital twins, based on mechanistic or hybrid deterministic stochastic models, that reproduce the manufacturing process in silico and predict the impact of variations in flow rate, mixing energy, temperature, or solvent composition on nanoparticle self-assembly, RNA integrity, and the thermodynamic and kinetic stability of the final drug [196,197].

The structural and compositional complexity of RNA-LNP systems also lends itself to digital and computational optimization. Machine learning models offer the possibility to identify sequence and structure-specific degradation hotspots, thereby informing the design of untranslated regions and other regulatory elements to improve resistance to nuclease-mediated and oxidative degradation while maintaining translational efficiency [198,199]. In parallel, computational screening of ionizable and helper lipid libraries can be used to prioritize chemistries with favorable pKa values, phase behavior, and endosomal escape properties prior to synthesis, reducing experimental burden and accelerating formulation development [147,200]. Within a Quality by Design (QbD) framework, these approaches support the concept of stability as an engineered attribute [201]. Predictive models trained on historical and real-time manufacturing and stability data can be used to delineate design spaces, estimate shelf-life under defined storage and distribution conditions, and guide adaptive control strategies during scale-up and throughout the product lifecycle [202,203].

A further priority is the development of robust in vitro–in vivo correlations (IVIVCs) that quantitatively link analytical markers to pharmacokinetic and pharmacodynamic outcomes. Future studies will need to define composite descriptors that integrate physicochemical and structural parameters such as size distribution, surface charge, apparent pKa, internal morphology, RNA integrity, and residual impurity profiles and correlate these with biodistribution, transgene expression kinetics, and immunogenicity in relevant preclinical and clinical models [204,205]. Once established and validated, such correlations could underpin the use of surrogate potency assays and reduce reliance on extensive animal studies for incremental formulation or process changes, while improving the predictive value of analytical release specifications for clinical performance [206,207].

Finally, the global scalability and long-term sustainability of RNA vaccine platforms will depend on the evolution of a harmonized and forward-looking regulatory framework. International coordination, particularly through entities such as the International Council for Harmonisation (ICH) and the World Health Organization (WHO), will be important to align expectations for stability study designs, impurity and potency acceptance criteria, and analytical requirements for emerging modalities, including saRNA and circular RNA [166,186]. The development of certified reference materials, such as RNA standards and well-characterized RNA-LNP panels, will facilitate inter-laboratory comparability and support convergence of analytical practices across regulatory jurisdictions [208].

## 12. Conclusions

RNA-based vaccines represent a transformative platform in modern pharmaceutical development. Their successful implementation depends on maintaining RNA integrity, optimizing formulation design, and applying robust analytical characterization strategies. Although significant progress has been achieved, challenges remain regarding long-term stability, method standardization, and regulatory harmonization. Future efforts should focus on integrated analytical approaches that establish stronger links between physicochemical properties and biological performance to support the development of safe, effective, and stable RNA-based vaccines.

## Figures and Tables

**Table 1:** Key platform differences between RNA-LNP and saRNA–LNP vaccines.

Dimension	RNA-LNP Vaccines	saRNA–LNP Vaccines
Expression mechanism	Direct translation of mRNA after cellular uptake	Intracellular self-amplification via a replicon system followed by antigen expression
RNA architecture	mRNA architecture regulated by UTRs to control stability and translation efficiency	Contains additional replicase sequences, increasing RNA length and enabling an intracellular amplification program
Dose requirements	Typically requires higher doses to achieve sufficient immune responses	Enables dose sparing due to intracellular self-amplification
Antigen expression duration and immune durability	Antigen expression duration and immune responses depend on construct and formulation design	Demonstrates prolonged antigen expression and sustained immune responses, maintaining antibody levels for extended periods
Physicochemical property–potency relationship	Physicochemical attributes alone do not guarantee functional potency, with the presence of critical thresholds for loss of efficacy	The same non-linear quality performance relationship applies to saRNA platforms

**Table 2:** Summary of analytical methods and corresponding quality attributes.

Analytical Technique	Corresponding Quality Attributes
EM/Cryo-TEM	Morphology and internal structure characterization of individual particles
DLS	Particle size, PDI, size distribution
ELS	Surface charge assessment
CDMS	Validation of empty RNA-LNP particle dimensions
TDA	Mass-weighted size distribution, PDI
Multi-detector AF4 (MD-AF4)	Subpopulation separation, aggregate detection
SEC-MALS/DLS	Separation-based size analysis
RiboGreen Fluorescence Assay	Bulk encapsulation efficiency measurement
AEX	mRNA subpopulation resolution; encapsulation heterogeneity assessment
IP-RP/IP-RPLC	Separation of adducts from intact mRNA; integrity assessment
2D-LC	Simultaneous EE, integrity, adduct, and transcript ratio data
SEC	Impurity and aggregate analysis
CICS	Single-particle analysis; empty RNA-LNP quantification
Technique	Corresponding quality attributes
CLiC-ALEX	mRNA distribution analysis; subpopulation characterization
Nanoflow Cytometry	Empty RNA-LNP detection; population heterogeneity quantification
CE/CGE	Detection of size-based mRNA degradation products
LC-MS/MS	mRNA sequence verification; chemical modification detection
Cell-Free Translation MS (CFT-MS)	Protein expression confirmation; frameshifting detection
NMR Spectroscopy	RNA-LNP chemical structure characterization
SANS/SAXS	RNA-LNP internal structural characterization
AUC	Stability indication; aggregate detection
iCIEF	Stability indication; charge variant analysis
LFSA	Lipid formulation stability monitoring

**Table 3:** Overview of potency assays and their applications for RNA-based vaccines.

Assay Category	Main Purpose	Assay Principle	Applications in RNA-Based Vaccines	Advantages	Limitations/Challenges	Suitable Use
CFT Assays: Cell-Free Translation Assays	Evaluates whether the RNA molecule can be translated into the intended protein antigen	Purified RNA is added to a cell-free translation system, such as lysate or recombinant translation machinery, and antigen production is measured	Early RNA construct screening, comparison of RNA sequence designs, assessment of RNA integrity after storage or stress, and confirmation that the RNA can produce an antigen	Rapid, simple, avoids cell-culture variability, useful for RNA-only functional testing	Does not measure cellular uptake, LNP delivery, endosomal escape, or immune-cell activation	Useful as an orthogonal functional assay, but usually not sufficient alone for final product potency
CBT Assays: Cell-Based Translation Assays	Determines whether RNA can enter cells and produce the encoded antigen	Cells are treated with RNA or RNA-LNP formulation, and antigen expression is measured inside or outside cells	Evaluation of mRNA-LNP delivery, translation efficiency, antigen expression, formulation comparison, dose–response testing	More biologically relevant than cell-free assays because cellular uptake and translation are included	Results depend on cell type, transfection efficiency, incubation time, detection method, and cell health	Suitable for potency testing, especially when linked to the mechanism of action
Cell-Based Potency Assays	Measures the biological activity of the complete RNA vaccine drug product	Vaccine formulation is applied to relevant cells, followed by measurement of antigen expression and/or downstream biological response	Lot release testing, stability testing, comparability studies, process-change evaluation, formulation-change evaluation	Directly evaluates the final formulated product and can reflect delivery plus translation	Requires a validated cell system, reference standard, assay controls, and tight variability control	Most relevant for release and stability-indicating potency assays
Functional Assays: Gain/Loss of Function	Confirms that the expressed antigen has the expected biological function or immune relevance	Cells expressing the antigen are tested for functional activity, receptor binding, neutralization sensitivity, immune recognition, or pathway activation	Antigen validation, functional confirmation of expressed protein, vaccine mechanism studies, comparison of variants or antigen designs	Provides biological meaning beyond simple protein expression	More complex, antigen-specific, may not be universal across vaccine platforms	Best for characterization, mechanism-of-action studies, and advanced potency support
Analytical/Quality Assays	Measures critical quality attributes that support potency and consistency	Uses physicochemical and molecular assays to evaluate RNA and nanoparticle quality	Batch characterization, release testing, impurity control, stability monitoring, and manufacturing consistency	Highly reproducible, quantitative, and useful for specifications and comparability	Analytical quality does not always predict biological potency by itself	Essential as supportive QC assays and used with functional bioassays
Formulation-Dependent Assays	Evaluates how formulation affects biological activity and stability	Tests RNA-LNPs under different lipid compositions, storage conditions, stress conditions, or delivery formats	LNP optimization, cold-chain assessment, lyophilization studies, shelf-life assignment, formulation comparability	Links nanoparticle structure to biological performance	Requires multiple orthogonal assays because formulation changes may affect size, stability, uptake, and translation differently	Important for formulation development, stability studies, and comparability testing

**Table 4:** RNA chemical stability vs. nanoparticle structural stability.

Feature	RNA Chemical Stability	Nanoparticle Structural Stability
Primary Nature	Chemical/covalent: Irreversible cleavage of phosphodiester bonds [9].	Physical/colloidal: Reversible or irreversible changes in size and morphology [128].
Main Mechanism	Hydrolysis & oxidation: Base-catalyzed hydrolysis (2′-OH attack) and oxidative base damage [121].	Aggregation & fusion: Increase in PDI and cargo leakage [119].
Key Influencing Factors	Enzymatic (RNases): High sensitivity to ubiquitous ribonucleases [9].	Thermodynamics: Lipid phase transitions (e.g., L_α_ to HII phase) and surface charge [119,120].
pH Sensitivity	Extremely sensitive to high pH (>8.0), which accelerates auto-hydrolysis [9,12].	Sensitive to pH fluctuations affecting the protonation of ionizable lipids [120].
Temperature Impact	Degradation follows the Arrhenius model; highly unstable at room temperature [121].	Freeze–thaw cycles can cause mechanical stress, leading to shell rupture [128].
Primary Stabilizers	Modified nucleosides (e.g., N1-methylpseudouridine) and 5′ capping [9,121].	PEGylated lipids (steric hindrance) and cholesterol (membrane rigidity) [119,120].
Storage Strategy	Use of DEPC-treated water and ultra-low temperature freezers (−80 °C) [9].	Lyophilization with cryoprotectants like sucrose or trehalose [128].

**Table 5:** Excipients and manufacturing parameters influencing RNA vaccine stability.

Component/Parameter	Category	Primary Role	Impact on Stability/Quality
Tris/Acetate Buffers	Excipient	Controls PH and stabilizes RNA integrity	Reduces hydrolytic degradation [155]
Sucrose/Trehalose	Excipient	Cryoprotectant	Inhibits aggregation of LNP during drying [160]
Ionizable Lipids	Excipient	Cargo Delivery	encapsulation efficiency, cellular uptake and efficiency of endosomal escape [154]
Mixing Speed (FRR)	Process	Kinetic Assembly	Regulates the size of LNP and PDI [55]
Lyophilization Cycle	Process	Thermostability	Improves long-term stability and enables refrigerated storage [161]
TFF	Process	Purification	Vital for purification and stress risks [116]
Shaking/Mixing Energy	Process	Mechanical Stress	Excessive shear or agitation disrupts LNP integrity and reduces encapsulation [157]
Digital Twins/AI	Advanced	Process Control	Real-time monitoring of CQAs [161]

## Data Availability

No new data were created or analyzed in this study. Data sharing is not applicable to this article.

## Abbreviations

The following abbreviations are used in this manuscript:

mRNA	Messenger RNA
saRNA	Self-Amplifying RNA
RNA-LNP	RNA-loaded Lipid Nanoparticle
CQAs	Critical Quality Attributes
UTRs	Untranslated Regions
PEG–lipid	Polyethylene Glycol Lipid
DLS	Dynamic Light Scattering
PDI	Polydispersity Index
Cryo-TEM	Cryogenic Transmission Electron Microscopy
EM	Electron Microscopy
ELS	Electrophoretic Light Scattering
CDMS	Charge Detection Mass Spectrometry
TDA	Taylor Dispersion Analysis
SEC	Size Exclusion Chromatography
SEC-MALS	Size Exclusion Chromatography–Multi-Angle Light Scattering
AEX	Anion Exchange Chromatography
IP-RP	Ion-Pair Reverse Phase
IP-RPLC	Ion-Pair Reversed-Phase Liquid Chromatography
2D-LC	Two-Dimensional Liquid Chromatography
CE	Capillary Electrophoresis
CGE	Capillary Gel Electrophoresis
LC-MS/MS	Liquid Chromatography–Tandem Mass Spectrometry
CFT-MS	Cell-Free Translation Mass Spectrometry
NMR	Nuclear Magnetic Resonance
SAXS	Small-Angle X-Ray Scattering
SANS	Small-Angle Neutron Scattering
AUC	Analytical Ultracentrifugation
iCIEF	Imaged Capillary Isoelectric Focusing
LFSA	Lipid Formulation Stability Assay
CICS	Cylindrical Illumination Confocal Spectroscopy
CLiC-ALEX	Convex Lens-Induced Confinement Alternating Laser Excitation
NTA	Nanoparticle Tracking Analysis
HPLC	High-Performance Liquid Chromatography
RT-PCR	Reverse Transcription Polymerase Chain Reaction
PCR	Polymerase Chain Reaction
qPCR	Quantitative Polymerase Chain Reaction
DEPC	Diethyl Pyrocarbonate
RNase	Ribonuclease
dsRNA	Double-Stranded RNA
PBS	Phosphate-Buffered Saline
PVP	Polyvinylpyrrolidone
API	Active Pharmaceutical Ingredient
ROS	Reactive Oxygen Species
TLR	Toll-Like Receptor
AREs	AU-rich Elements
PABP	Poly(A)-Binding Protein
ED50	Effective Dose 50
CBT	Cell-Based Translation
CFT	Cell-Free Translation
DC	Dendritic Cell
MD	Molecular Dynamics
RSV	Respiratory Syncytial Virus
ICH	International Council for Harmonisation
LAL	Limulus Amebocyte Lysate
BioRender	Biological Illustration Software Platform
